# The interactome of metabolic enzyme carbonic anhydrase IX reveals novel roles in tumor cell migration and invadopodia/MMP14-mediated invasion

**DOI:** 10.1038/onc.2017.219

**Published:** 2017-07-10

**Authors:** M Swayampakula, P C McDonald, M Vallejo, E Coyaud, S C Chafe, A Westerback, G Venkateswaran, J Shankar, G Gao, E M N Laurent, Y Lou, K L Bennewith, C T Supuran, I R Nabi, B Raught, S Dedhar

**Affiliations:** 1 Department of Integrative Oncology, BC Cancer Research Centre, Vancouver, British Columbia, Canada; 2 Department of Biochemistry and Molecular Biology, University of British Columbia, Vancouver, British Columbia, Canada; 3 Princess Margaret Cancer Centre, University Health Network, Toronto, Ontario, Canada; 4 Department of Cellular and Physiological Sciences, University of British Columbia, Vancouver, British Columbia, Canada; 5 Laboratorio di Chimica Bioinorganica, Universita degli Studi di Firenze, Sesto Fiorentino, Florence, Italy

## Abstract

Carbonic anhydrase IX (CAIX) is a hypoxia inducible factor 1-induced, cell surface pH regulating enzyme with an established role in tumor progression and clinical outcome. However, the molecular basis of CAIX-mediated tumor progression remains unclear. Here, we have utilized proximity dependent biotinylation (BioID) to map the CAIX ‘interactome’ in breast cancer cells in order to identify physiologically relevant CAIX-associating proteins with potential roles in tumor progression. High confidence proteins identified include metabolic transporters, β1 integrins, integrin-associated protein CD98hc and matrix metalloprotease 14 (MMP14). Biochemical studies validate the association of CAIX with α2β1 integrin, CD98hc and MMP14, and immunofluorescence microscopy demonstrates colocalization of CAIX with α2β1 integrin and MMP14 in F-actin/cofilin-positive lamellipodia/pseudopodia, and with MMP14 to cortactin/Tks5-positive invadopodia. Modulation of CAIX expression and activity results in significant changes in cell migration, collagen degradation and invasion. Mechanistically, we demonstrate that CAIX associates with MMP14 through potential phosphorylation residues within its intracellular domain, and that CAIX enhances MMP14-mediated collagen degradation by directly contributing hydrogen ions required for MMP14 catalytic activity. These findings establish hypoxia-induced CAIX as a novel metabolic component of cellular migration and invasion structures, and provide new mechanistic insights into its role in tumor cell biology.

## Introduction

Tumor cell migration and invasion contribute significantly to the formation of metastases, which are responsible for tumor-associated mortality.^1^ The mechanisms involved in these processes are complex and are modulated by many genetic and microenvironmental factors. Tumor hypoxia is a significant component of the microenvironment of most solid tumors, and it is known to promote epithelial–mesenchymal transition, tumor cell migration and invasion.^2^

The inhibition of oxidative phosphorylation in hypoxia is accompanied by an increase in glycolytic metabolism, resulting in accumulation and subsequent extrusion of lactate by cancer cells into the tumor microenvironment, leading to acidic extracellular pH (pHe). A major consequence of extracellular acidosis is the disruption of intracellular pH (pHi) homeostasis, the maintenance of which is essential for a spectrum of critical cellular functions. Tumor cells adapt to the harsh conditions imposed by hypoxia and acidosis by activating an efficient pH regulatory system to prevent intracellular acidification. In addition to increasing the activity of sodium-hydrogen exchanger-1 (NHE1), a ubiquitously expressed regulator of pH homeostasis,^3^ cancer cells upregulate carbonic anhydrase IX (CAIX), a hypoxia-induced cell surface protein that regulates pHi and promotes tumor cell survival.^4, 5^ A major consequence of pHi regulation is an increasingly acidic pHe, which has been shown to activate proteases and stimulate local matrix degradation and tissue remodeling.^6, 7^ Recent studies have demonstrated a critical role of CAIX in tumor growth and metastasis,^8, 9, 10, 11^ and while CAIX has been suggested to play a role in tumor invasion,^12, 13^ the molecular basis of CAIX-mediated motility and invasion remains poorly understood.

Although tumor hypoxia and acidosis stimulate tumor cell migration and invasion, the role of hypoxia in the formation and function of pseudopodia, a broad term defining cytoplasmic extensions of lamellar (lamellipodia, ruffles), filamentous (filopodia) or spherical (blebs) shape,^14^ and invadopodia, defined as protrusive structures enriched in actin and actin regulators such as integrins, cortactin, the Wiskott–Aldrich syndrome protein N-WASp, Arp2/3, cofilin and Tks5^15^ that function to degrade extracellular matrix,^16^ has only been explored recently.^17, 18^ In particular, tumor cell invasion is facilitated by the formation of invadopodia. The maturation of invadopodia involves talin-mediated recruitment of NHE1,^19^ which plays a critical role in regulating invadopodia and pseudopodia function by modulating pHi^20, 21^ and drives cofilin-dependent actin polymerization and recruitment of matrix metalloproteases (MMPs) such as MMP14 (also known as membrane-type 1-MMP; MT1-MMP).^19^ As a consequence of its function in regulating pHi at invadopodia, NHE1 extrudes protons (H^+^) into the extracellular environment, thereby contributing to extracellular acidosis. However, hypoxia has been shown to inhibit NHE1 activity^22^ and recent interrogation of The Cancer Genome Atlas (TCGA) for *NHE1* gene expression in primary breast tumor samples has shown that *NHE1* gene expression is significantly lower in tumors of the basal subtype compared to luminal and human epidermal growth factor receptor 2-positive (HER2^+^) subtypes,^23^ suggesting the importance of pH regulatory proteins such as CAIX in contributing to extracellular acidosis in hypoxia. Invadopodia concentrate proteases such as MMP14, MMP9 and MMP2 for local directed release during extracellular matrix breakdown, and along with cortactin and Tks5, have now been shown to be required for tumor cell extravasation during metastasis.^24^

While previous studies have shown that hypoxia potentiates the formation of invadopodia by cancer cells in a hypoxia inducible factor 1 alpha (HIF-1α)-dependent manner^25^ through the regulation of growth factor pathways and the expression of proteases such as MMP14,^26^ the potential role of CAIX in this process has not been examined. Here, we demonstrate that CAIX plays a critical role in tumor cell migration, invasion and metastasis. Utilizing an unbiased proximity ligation (BioID) approach, we have mapped the CAIX interactome, and find that CAIX associates not only with several cell surface metabolic transport proteins, but also with β1 integrins, especially the collagen and laminin receptors, α2/β1, α3/β1 and α6/β1, and, interestingly, the matrix metalloprotease, MMP14. In addition, CAIX associates with cluster of differentiation 98 heavy chain (CD98hc), encoded by the solute carrier family member (SLC) 3A2 (*SLC3A2*) gene, a component of an amino acid transporter that has been shown to associate with β1 integrin and mediate its signalling.^27^ Integrins and MMP14 are components of pseudopodia and invadopodia, and we find that CAIX colocalizes with F-actin, cofilin, α2/β1 integrin and MMP14, specifically in pseudopodia-like protrusions resembling lamellipodia, but not in focal adhesion kinase (FAK)-positive focal adhesions when plated on type 1 collagen. CAIX also colocalizes with cortactin, Tks5 and MMP14-positive mature invadopodia. Finally, we demonstrate that CAIX associates with MMP14 through its intracellular domain, and that CAIX catalytic activity is required for the stimulation of MMP14-mediated collagen degradation through direct hydrogen ion (H^+^)-mediated stimulation of MMP14 activity.

## Results

### Proximity-dependent biotinylation (BioID) analysis identifies a complex CAIX interactome in cancer cells with roles in pH regulation, metabolite transport and tumor cell migration and invasion

**Figure 1:**
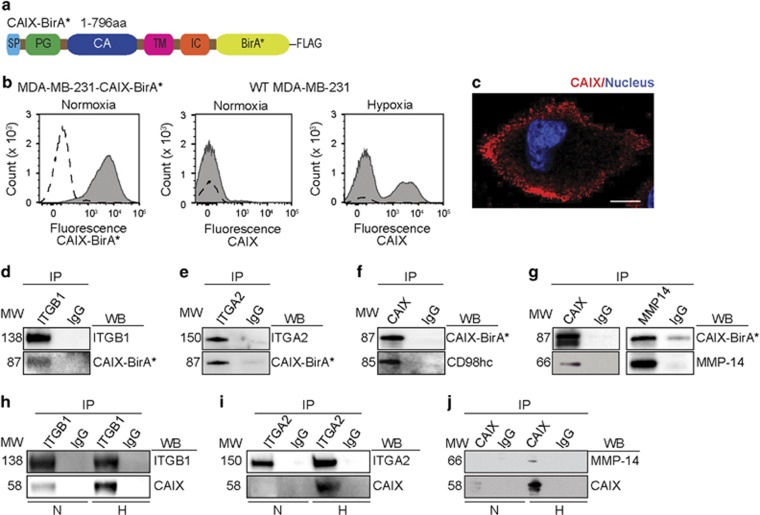
Identification of the CAIX Interactome using the BioID proximal ligation strategy and validation of high confidence proximal CAIX-associating proteins by co-immunoprecipitation. (**a**) Schematic showing the domain structure of the CAIX-BirA* fusion protein used for the BioID studies. SP, signal peptide; PG, proteoglycan-like domain; CA, catalytic domain; TM, transmembrane domain; IC, intracellular domain. (**b**) FACS analysis showing levels of constitutive expression of the CAIX-BirA* fusion protein expressed by MDA-MB-231 cells (MDA-MB-231-CAIX-BirA*) cultured in normoxia compared to levels of endogenous CAIX expression by WT MDA-MB-231 cells cultured in either normoxia or hypoxia. The filled (gray) histograms indicate specific CAIX staining whereas the dotted lined histograms indicate staining by isotype-specific control IgG. (**c**) Image showing exogenous CAIX (red) localized at the cell surface of MDA-MB-231-CAIX-BirA* cells grown in normoxia. Scale bar, 10 μM. (**d**–**g**) Co-IP of high confidence proximal CAIX-associating proteins (**d**) ITGB1, (**e**) ITGA2 (**f**) CD98hc and (**g**) MMP14 with exogenous CAIX expressed by MDA-MB-231-CAIX-BirA* cells cultured in normoxia. (**h**–**j**) Co-IP of (**h**) ITGB1, (**i**) ITGA2 and (**j**) MMP14 with endogenous CAIX expressed by WT MDA-MB-231 cells cultured in either normoxia (N) or hypoxia (H). Isotype-specific IgG was used as a control for co-IPs and data in (**d**) to (**j**) are representative of at least two independent experiments. IgG, immunoglobulin G.

CAIX is widely regarded as a prominent biomarker of poor patient prognosis and treatment resistance, yet the identities of physiologically and functionally relevant CAIX associated proteins in cancer cells remain unknown. Here, we utilized a proximity-dependent biotinylation (BioID) proteomic approach^28^ to identify CAIX-associating partners *in situ* in cancer cells. To conduct these studies, we first coupled biotin ligase (BirA*) to the C-terminus of full length, wild-type (WT) human *CA9* (Figure 1a) and expressed the CAIX-BirA* fusion protein constitutively in WT MDA-MB-231 human triple negative breast cancer cells (MDA-MB-231-CAIX-BirA*). Expression of BirA* alone (MDA-MB-231-BirA*) provided a control for binding specificity. Fluorescence activated cell sorting (FACs) analysis demonstrated that the level of CAIX-BirA* fusion protein expressed constitutively at the surface of cells grown in normoxia was approximately two-fold higher when compared to the level of expression of endogenous CAIX by cells cultured in hypoxia (Figure 1b), demonstrating the presence of robust levels of protein expression necessary for successful application of the BioID approach. Appropriate localization of exogenously expressed CAIX at the cell surface was further verified by immunofluorescence microscopy (Figure 1c). To perform the BioID analysis, MDA-MB-231-CAIX-BirA* cells and MDA-MB-231-BirA* control cells were cultured in either normoxia or hypoxia, followed by addition of culture media containing biotin and incubation for a further 24 h to induce labeling of cellular proteins. Cells were then collected, labeled proteins were enriched via streptavidin-Sepharose and analysis was carried out by mass spectrometry.^29^ Two replicates of MDA-MB-231-CAIX-BirA* cells were compared to 4 runs of MDA-MB-231-BirA* control cells and a set of 16 generic control runs (2 runs of no bait controls from MCF7 cells, 4 runs of BirA* alone expressed in MCF10A cells, and 10 runs of BirA* alone expressed in HEK293 T-REx cells). Control data were collapsed to the 2 highest spectral counts for each detected prey protein. A Significance Analysis of INTeractome (SAINT) Bayesian false discovery rate ⩽0.01^30^ was employed as a threshold and used to generate an initial set of high confidence proximal proteins associating with CAIX (Supplementary Table S1).

Interrogation of these proteomic data revealed robust associations between CAIX and several classes of cell surface proteins, particularly transport proteins with roles in metabolism and metabolic regulation of pH (Table 1), and proteins linked to adhesion, migration and invasion (Table 1). The most prevalent transporters identified include the amino acid transport protein heavy chain SLC3A2 (CD98hc), the glutamine transporter, SLC38A2, the bicarbonate (HCO_3_^−^) transporter, SLC4A7, known to couple with CAIX for the transport of HCO_3_^−^ into cells to neutralize acidic pHi in hypoxia,^31^ and the fatty acid transporter, SLC27A4. Among the CAIX-associating proteins involved in adhesion, migration and invasion, the integrin subunits integrin β1 (ITGB1), integrin α2 (ITGA2), integrin α3 (ITGA3) and integrin α6 (ITGA6) were strongly represented, as was MMP14 (MT1-MMP), demonstrating the potential association of CAIX with collagen and laminin integrins, and membrane-localized MMPs.

Owing to the well-recognized role of integrins and MMP14 in cell migration and invasion, coupled with previous data demonstrating the importance of CAIX in promoting the growth and metastasis of solid tumors,^8, 9, 10, 11^ we were particularly interested in the strong association of CAIX with these proteins. Thus, we focused our attention on validating the observed associations between CAIX and integrins, CD98hc, and MMP14 by co-immunoprecipitation (co-IP). Given that the proteins identified using the BioID approach were found to associate with exogenously expressed CAIX equally well in normoxia and hypoxia (Supplementary Table S1), initial validation was performed using MDA-MB-231-CAIX-BirA* cells. We observed that ITGB1 (Figure 1d, Supplementary Figure S1a), ITGA2 (Figure 1e, Supplementary Figure S1a), CD98hc (Figure 1f, Supplementary Figure S1a) and MMP14 (Figure 1g, Supplementary Figure S1a) readily co-IPed with exogenously expressed CAIX. In addition, the association between MMP14 and CAIX was further confirmed by reciprocal co-IP strategies (Figure 1g). Together, these results validate the presence of associations between CAIX, integrins and MMP14.

Importantly, while the co-IP data clearly demonstrate that integrins and MMP14 associate with CAIX in cells constitutively expressing CAIX, the data do not address whether these proteins associate with endogenous CAIX or whether the associations are present in hypoxia, a biologically relevant tumor environmental context. Indeed, CAIX is strongly upregulated by WT MDA-MB-231 cells cultured in hypoxia (Supplementary Figure S1b), and, as such, we hypothesized that increased abundance of CAIX in hypoxia may result in a parallel increase in the amount of CAIX associating with these proteins. To test this idea, WT MDA-MB-231 cells were cultured in hypoxia to induce HIF-1-mediated upregulation of CAIX and co-IP experiments were performed. We observed that large amounts of CAIX co-IPed with ITGB1, ITGA2 and MMP14 from cells cultured in hypoxia, whereas much less CAIX co-IPed with these proteins in similar cells cultured in normoxia (Figures 1h–j, Supplementary Figure S1b). These results validate the association of integrin α2/β1 and MMP14 with endogenous CAIX expressed by human breast cancer cells, and demonstrate that the upregulation of CAIX in hypoxia is mirrored by an increase in quantity of CAIX associating with these proteins.

To extend these findings, we conducted similar experiments using PK-8 human pancreatic ductal adenocarcinoma cells grown in normoxia and hypoxia. Corroborating our findings in the breast cancer cells, CAIX expression was upregulated by PK-8 pancreatic ductal adenocarcinoma cells cultured in hypoxia and CAIX co-IPed with MMP14 in a reciprocal manner from these cells, whereas the association was not readily detected in PK-8 cells grown in normoxia (Supplementary Figures S1c and d). Taken together, these data demonstrate that CAIX associates with integrins and MMP14 in cancer cells, that these associations are present in the environmental context of hypoxia, and that the amount of CAIX associating with these proteins is congruent with the levels of hypoxia-induced expression of CAIX.

### CAIX colocalizes with integrins and MMP14 in pseudopodia-like protrusions resembling lammelipodia and regulates tumor cell migration

**Figure 2:**
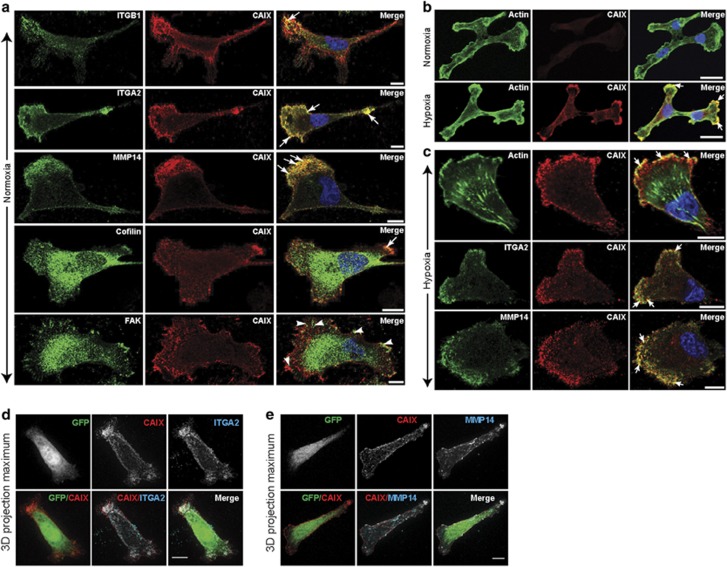
CAIX colocalizes with integrins and MMP14 in pseudopodia-like protrusions resembling lamellipodia. (**a**) Images of MDA-MB-231-CAIX-BirA* cells cultured on type 1 collagen in normoxia, showing colocalization (yellow, arrows in merge) of exogenously expressed CAIX (red) with ITGB1, ITGA2, MMP14 and cofilin (each marker in green), but not with FAK (green; arrowheads). Scale bar, 10 μm. (**b**) Images of WT MDA-MB-231 cells grown in either normoxia or hypoxia, showing colocalization of hypoxia-induced CAIX (red) with F-actin (green) in pseudopodia-like protrusions (arrows, merge). Scale bar, 10 μm. (**c**) Images of WT MDA-MB-231 cells cultured on type 1 collagen in hypoxia, showing colocalization of hypoxia-induced CAIX (red) with F-actin, ITGA2 and MMP14 (green) at leading edges of cells (arrows, merge), but not at focal adhesions. Scale bar, 10 μm. (**d** and **e**) 3D maximum projections of 2D images of MDA-MB-231-CAIX-BirA* cells transfected with GFP and cultured on type 1 collagen in normoxia, showing robust colocalization of CAIX (red) with (**d**) ITGA2 and (**e**) MMP14 (blue) in pseudopodia-like protrusions and depletion of cytoplasmic GFP (green) from these regions. Images for each channel are shown in the upper panels and merged images are shown in the lower panels. Scale bar, 10 μm.

The data captured by the BioID proteomic analysis and subsequent validation of key associations by co-IP analyses suggest that CAIX associates strongly with several proteins involved in adhesion, migration and invasion, particularly ITGB1, ITGA2 and MMP14. These findings propelled us to investigate whether CAIX colocalizes with these proteins within specific regions in breast cancer cells, especially at membrane protrusions typical of migrating cells. Congruent with the co-IP studies described above, we initially evaluated the colocalization of CAIX with ITGB1, ITGA2 and MMP14 in the MDA-MB-231-CAIX-BirA* cells. Cells were cultured on type I collagen, a relevant substrate for studies involving localization of collagen-binding integrins and MMP14. Interestingly, we observed that CAIX colocalizes specifically with ITGB1, ITGA2 and MMP14 in actin- and cofilin-positive pseudopodia-like protrusions resembling lamellipodia, but not with ITGB1 or FAK located at focal adhesions (Figure 2a), suggesting that CAIX associates with these proteins specifically in cellular regions involved in migration.

To confirm and strengthen these findings, we conducted further experiments to examine the localization of hypoxia-induced CAIX at similar protrusions in WT MDA-MB-231 cells. First, we cultured these cells in normoxia or hypoxia and evaluated the presence and localization of CAIX expression relative to the expression of actin. As expected, actin was expressed at similar levels by cells cultured in either oxygen (O_2)_ tension and was strongly expressed in pseudopodia-like protrusions (Figure 2b). In contrast, CAIX was expressed at very low basal levels in normoxia, but was strongly induced by cells cultured in hypoxia (Figure 2b). Furthermore, hypoxia-induced CAIX was locally concentrated at pseudopodia-like protrusions and colocalized with actin in these regions (Figure 2b). Based on these data, we performed further studies using cells cultured on type 1 collagen specifically in hypoxia to ensure the presence of abundant amounts of CAIX. We observed that hypoxia-induced CAIX clearly colocalizes with actin, ITGA2 and MMP14 at pseudopodia-like protrusions resembling lamellipodia, but not at focal adhesions, at the leading edges of cells displaying a migratory phenotype (Figure 2c). Together with the data demonstrating co-IP of CAIX with these proteins, the observations of colocalization suggest that the association of CAIX with integrins and MMP14 play a potential role in the regulation of breast tumor cell migration.

To mitigate the possibility that the colocalization of CAIX with integrins and MMP14 at pseudopodia-like protrusions may be the result of volume-related effects in the protrusions relative to the cytoplasm, we cultured MDA-MB-231-CAIX-BirA* cells on collagen in normoxia, transfected the cells with green fluorescent protein (GFP) as a freely distributing marker of the cytoplasm, and assessed the localization of all markers using three dimensional (3D) maximum projections. We observed that whereas CAIX and ITG2A clearly colocalize and are enriched in pseudopodia-like protrusions resembling lamellipodia, GFP localizes to the cytoplasm and is depleted from these protrusions (Figure 2d). Similarly, CAIX and MMP14 colocalize and concentrate at cellular protrusions (Figure 2e), whereas GFP localizes largely to cytoplasm outside these regions. Taken together, these data reinforce the findings that CAIX colocalizes with integrins and MMP14 specifically at protrusive structures and that the observations are not the result of volume-related effects.

**Figure 3:**
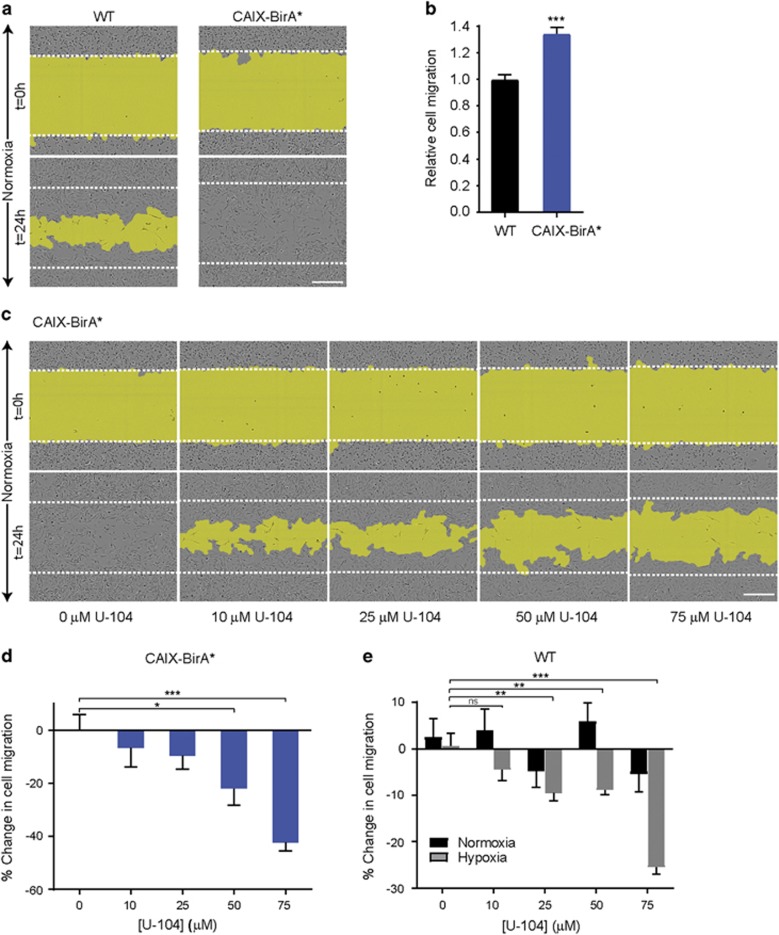
CAIX regulates migration of breast cancer cells. (**a**) Images of wound-induced cell migration by the indicated MDA-MB-231 cells cultured in normoxia. Scale bar, 300 μm. Dashed lines demarcate the wound boundary at *t*=0 h and yellow shading indicates the cell-free area. (**b**) Quantification of migration by the cells described in **a**. ***P<0.001. (**c**) Images of wound-induced migration of MDA-MB-231 CAIX-BirA* cells cultured in normoxia and treated with increasing concentrations of U-104. Scale bar, 300 μm. Wound boundaries and cell-free area are demarcated as described in **a**. (**d**) Quantification of migration by the cells described in **c**. *P<0.05, ***P<0.001. Data in panels (**b**) and (**d**) show the mean±s.e.m. of technical replicates (*n*=16) and are representative of two independent experiments. (**e**) Wound-induced migration of WT MDA-MB-231 cells cultured in normoxia (black bars) or hypoxia (gray bars) and treated with U-104. Data show the mean±s.e.m. of technical replicates (*n*=12) and are representative of three independent experiments. **P<0.01, ***P<0.001. Statistical analysis was performed using Student’s *t*-test (**b**) or ANOVA (**d**,**e**).

Prompted by our findings demonstrating clear colocalization of CAIX with integrins and MMP14 at the leading edges of cells, we performed chemotaxis and wound-induced migration assays to investigate the functional contribution of CAIX to the migration of breast cancer cells. Expression of CAIX by MDA-MB-231-CAIX-BirA* cells cultured in normoxia significantly enhanced both wound-induced migration (Figures 3a and b) and epidermal growth factor-induced migration (Supplementary Figure S2a) when compared to control cells. Furthermore, treatment of these cells with a specific small molecule inhibitor of CAIX activity, U-104,^9, 32^ significantly and dose-dependently inhibited wound-induced migration (Figures 3c and d, Supplementary Movies S1 and 2) and reduced epidermal growth factor-stimulated migration (Supplementary Figure S2b) compared to vehicle-treated cells. Similar treatment of WT MDA-MB-231 cells grown in normoxia, conditions in which CAIX is not induced, had no significant effect (Supplementary Figure S2c).

To investigate the effect of inhibiting CAIX activity on cell migration in the context of hypoxia-induced CAIX expression, wound-induced migration assays were carried out using WT MDA-MB-231 cells. Treatment of cells cultured in hypoxia with increasing concentrations of U-104 resulted in a significant, dose-dependent reduction in migration, while there was no significant effect of treatment on cells grown in normoxia (Figure 3e), in line with basal levels of CAIX expression in conditions of ambient O_2_. Taken together, these data demonstrate a potent effect of CAIX on migration of breast tumor cells and suggest that the catalytic activity of CAIX is important for the regulation of cell migration.

### CAIX colocalizes with MMP14 in mature invadopodia and regulates MMP14-mediated type I collagen degradation and invasion

**Figure 4:**
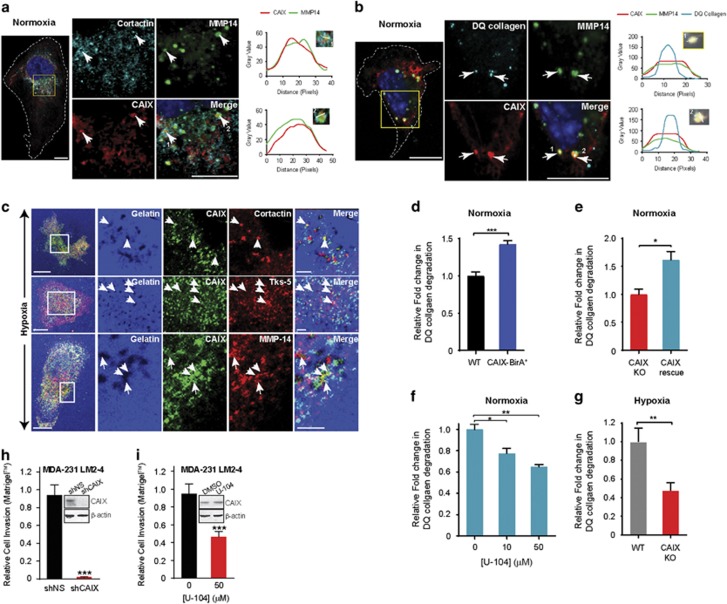
CAIX colocalizes with MMP14 at invadopodia and regulates type I collagen degradation. (**a**) Images of a MDA-MB-231-CAIX-BirA* cell (white dashed line shows boundary) cultured on type I collagen in normoxia, identifying a ROI (yellow box) containing invadopodia (arrows) labeled for Cortactin (cyan), CAIX (red) and MMP14 (green). Profile plots showing colocalization of CAIX and MMP14 at cortactin-positive invadopodia (1 and 2) along the indicated lines (insets) are shown at the right. Scale bar, 10 μm. (**b**) Images of an MDA-MB-231-CAIX-BirA* cell (white dashed line shows cell boundary) cultured on DQ type I collagen in normoxia, identifying an ROI (yellow box) containing regions of collagen degradation (1 and 2, arrows) that are labeled for DQ collagen (cyan), CAIX (red) and MMP14 (green). Profile plots showing colocalization of the signals along the indicated lines (insets) are shown to the right. Scale bar, 10 μm. (**c**) Images showing WT MDA-MB-231 cells cultured in hypoxia on fluorescently labeled gelatin (blue, with black areas denoting regions of degradation) and stained for CAIX (green) and markers of invadopodia (red), including cortactin, Tks5 and MMP14 to demonstrate colocalization at mature invadopodia (white arrows; yellow foci). Scale bars, 10 μm (left), 2 μm (right). (**d**) Quantification of DQ collagen degradation by the indicated MDA-MB-231 cell lines grown in normoxia. ***P<0.001. (**e**) Quantitation of degradation of DQ collagen by MDA-MB-231 CAIX KO and CAIX rescue cells cultured in normoxia. *P<0.05. Data in (**d**) and (**e**) show the mean±s.e.m. of technical replicates (*n*=5) and are representative of three independent experiments. (**f**) Dose-dependent inhibition of DQ collagen degradation by CAIX rescue cells treated with U-104. Data show the mean±s.e.m. of technical replicates (*n*=4) and are representative of two independent experiments. *P<0.05, **P<0.01. (**g**) Quantification of DQ collagen degradation by WT MDA-MB-231 and CAIX KO cells cultured in hypoxia. Data show the mean±s.e.m. of technical replicates (*n*=8) and are representative of three independent experiments. **P<0.01. (**h** and **i**) Invasion through Matrigel by highly metastatic MDA-MB-231 LM2-4 breast cancer cells cultured in hypoxia and (**h**) expressing CAIX-targeted (shCAIX) shRNA or (**i**) treated with U-104. Data show the mean±s.e.m. of three independent experiments. ***P<0.001. Western blots of hypoxia-induced CAIX expression are shown as insets.

In addition to its localization to cellular structures involved in migration, MMP14 is a known component of invadopodia where it is involved in matrix degradation. In light of the data shown here demonstrating that CAIX co-IPs and colocalizes with MMP14, we next investigated CAIX as a component of these invasive structures. Indeed, in experiments using MDA-MB-231-CAIX-BirA* cells cultured on collagen, we observed that CAIX colocalizes with MMP14 to discrete puncta that are positive for cortactin, a prototypic marker of invadopodia (Figure 4a), as well as at sites of collagen degradation (Figure 4b), suggesting that CAIX and MMP14 associate at mature, active invadopodia.

To extend these findings, we investigated whether CAIX and MMP14 also colocalized at invadopodia in the context of hypoxia-induced CAIX expression. WT MDA-MB-231 cells were cultured on fluorescent gelatin in hypoxia, a collagen substrate that allows for identification of functional invadopodia based on production of ‘cleared areas’ in the fluorescent substrate. We observed that hypoxia-induced CAIX clearly colocalizes with multiple markers of invadopodia, including cortactin andTks5, as well as with MMP14 at mature, active invadopodia in these cells (Figure 4c). Colocalization of CAIX to invadopodia is also demonstrated in 4T1 cells, a murine model of triple negative breast cancer, grown in hypoxia (Supplementary Figures S3a–d).

Our findings demonstrating the colocalization of MMP14 and CAIX at mature invadopodia suggested to us that CAIX plays an active role in collagen degradation, an essential process for cell invasion. To further investigate this avenue, we developed a quantifiable collagen degradation assay. We observed that collagen degradation by MDA-MB-231-CAIX-BirA* cells is significantly enhanced relative to degradation by WT MDA-MB-231 cells grown in normoxia (Figure 4d).

To confirm these data, we generated two additional MDA-MB-231 cell lines, one in which the *CA9* gene was deleted using Clustered regularly interspaced short palindromic repeats (CRISPR)-Cas9 gene editing technology (CAIX knockout (KO), Supplementary Figure S4a) and one in which human *CA9* was re-introduced (CAIX rescue). Western blot and FACS analyses confirmed complete absence of hypoxia-induced CAIX expression in the CAIX KO cells and constitutive expression of CAIX by the CAIX rescue cells (Supplementary Figures S4b and c). Similar to the results using MDA-MB-231-CAIX-BirA* cells, collagen degradation by the MDA-MB-231 CAIX rescue cells was significantly increased compared to MDA-MB-231 CAIX KO cells (Figure 4e). Furthermore, treatment of the CAIX rescue cells with U-104 resulted in dose-dependent inhibition of collagen degradation (Figure 4f), while treatment of CAIX KO cells had no effect (Supplementary Figure S4d). In addition, while WT MDA-MB-231 cells cultured in hypoxia actively degraded collagen, this activity was significantly inhibited in the CAIX KO cells (Figure 4g), suggesting that CAIX is required for collagen degradation in hypoxia. There was no significant difference between the two cell lines when cultured in normoxia (Supplementary Figure S4e).

Having demonstrated that CAIX contributes the process of collagen degradation, we next sought to determine the effect of CAIX on cell invasion. Analysis of invasion through Matrigel by highly metastatic MDA-MB-231 LM2-4 human breast cancer cells cultured in hypoxia demonstrated significant inhibition of invasion by either shRNA-mediated silencing of CAIX (Figure 4h), or inhibition of its activity with U-104 (Figure 4i). Under the conditions of these assays, there was no significant effect on cell death (Supplementary Figure S4f).

### CAIX is required for breast tumor cell invasion and metastasis

**Figure 5:**
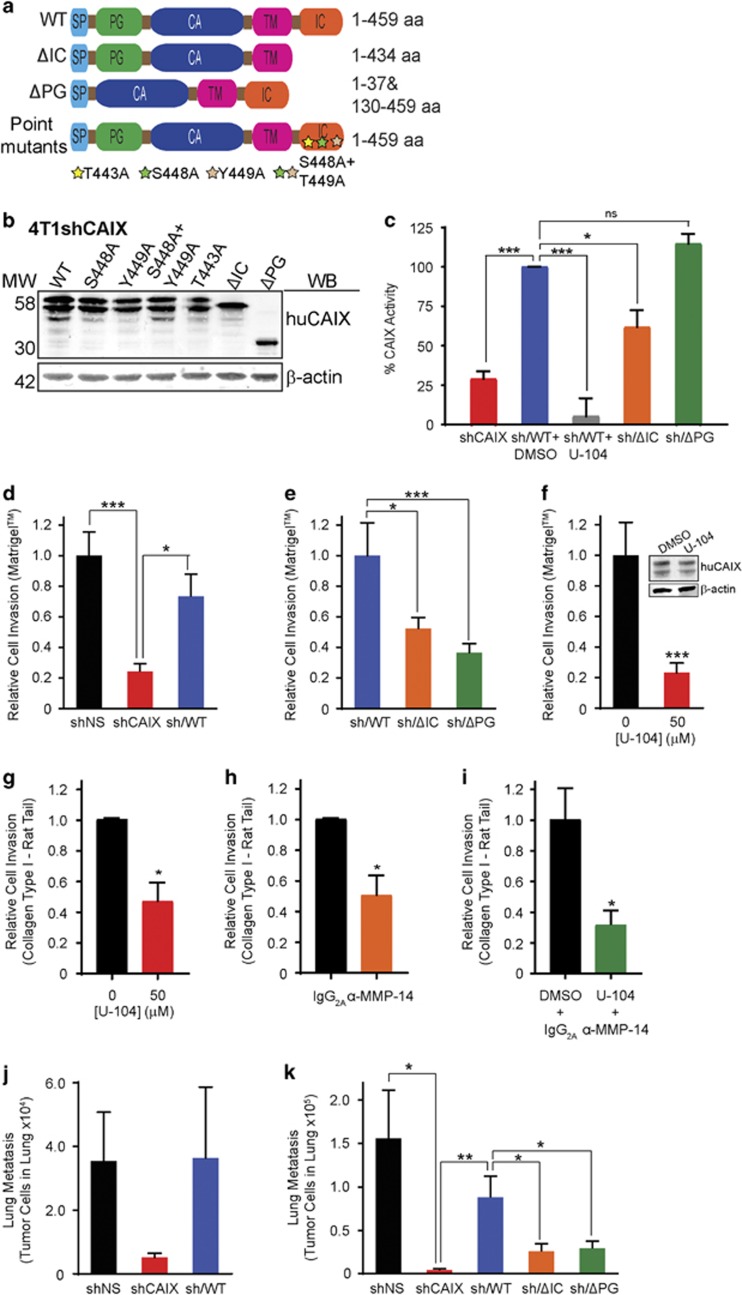
CAIX regulates breast cancer cell invasion and metastasis. (**a**) Schematic showing the domain structure of wild type (WT) huCAIX (459 aa) and constructs lacking either the intracellular domain (ΔIC), the extracellular proteoglycan-like domain (ΔPG) or the indicated point mutations in the IC domain. SP, signal peptide; CA, catalytic domain; TM, transmembrane domain. (**b**) Western blot analysis of the levels of expression of the indicated huCAIX constructs by 4T1shCAIX cells cultured in hypoxia. β-actin served as a loading control. (**c**) Analysis of CAIX catalytic activity using the *in-cell* carbonic anhydrase activity assay. Assays were performed in normoxia in the presence or absence of U-104 (50 μM) as indicated. Levels of CAIX catalytic activity were normalized by using the time required to achieve 50% of the total decrease in pH. Data were normalized to the spontaneous rate of reaction in the presence of buffer alone and the activity of cells expressing WT huCAIX was set to 100%. Data show the mean±s.e.m. of technical replicates (*n*=3/group) and are representative of three independent experiments *P<0.05, ***P<0.001. (**d**) Invasion through Matrigel by the indicated 4T1 cell lines cultured in hypoxia. Data show the mean±s.e.m. of three independent experiments. *P<0.05, ***P<0.001. (**e**) Invasion through Matrigel by the 4T1shCAIX cell lines expressing WT, ΔIC and ΔPG variants of huCAIX and cultured in hypoxia. *P<0.05, ***P<0.001. (**f**) Invasion through Matrigel by 4T1 sh/WT huCAIX cells cultured in hypoxia and treated with U-104. ***P<0.001. Western blots of hypoxia-induced CAIX expression are shown as insets. For (**d**)– (**f**), data show the mean±s.e.m. of three independent experiments. (**g**–**i**) Analysis of invasion through type 1 collagen by 4T1 sh/WT huCAIX cells cultured in hypoxia and treated with (**g**) U-104 (50 μM), (**h**) anti-MMP14 antibody (20 μg/ml) or (**i**) a combination of anti-MMP14 antibody (20 μg/ml) and U-104 (50 μM). *P<0.05, **P<0.01. Data in (**g**)–(**i**) show the mean±s.e.m. of three independent experiments. (**j**) Analysis of spontaneous lung metastases formed by the indicated 4T1 cell lines following growth of orthotopic breast tumors. Data show the mean±s.e.m. *n*=6/group. (**k**) Analysis of experimental lung metastases formed by the indicated 4T1 cell lines. Mean±s.e.m. is shown. *n*=6/group. *P<0.05, **P<0.01. Statistical analysis was performed using ANOVA (**c**,**d**,**e**,**k**) or Student’s *t*-test (**f**,**g**,**h**).

Having established that CAIX and MMP14 colocalize at mature invadopodia, and that CAIX regulates collagen degradation and invasion, we wanted to gain further insight into the mechanisms underlying the CAIX-MMP14 association and its role in cell invasion and metastasis. We utilized the 4T1 murine breast cancer model in which we have demonstrated a critical role of CAIX in metastasis formation.^9^ Importantly, the 4T1 cell line is a well-established, tractable syngeneic model of CAIX-positive metastatic breast cancer and offers the distinct advantage of allowing for shRNA-mediated depletion of endogenous murine CAIX in combination with constitutive expression of human CAIX, which is resistant to shRNA directed toward the murine isoform. Thus, using WT human CAIX (huCAIX) as a template, we engineered truncated variants of huCAIX lacking either the intracellular (IC) domain (ΔIC) or the proteoglycan-like (PG) domain (ΔPG), as well as single and double point mutations at putative phosphorylation sites within the IC domain, including threonine 443 to alanine (T443A), serine 448 to alanine (S448A), tyrosine 449 to alanine (Y449A) and S448A+Y449A (Figure 5a, Supplementary Figure S5a). Then, using 4T1 cells stably depleted of murine CAIX expression (4T1shCAIX) (Supplementary Figure S5b), we generated cell lines constitutively expressing WT huCAIX (designated 4T1sh/WT) (Figure 5b, Supplementary Figure S5b), as well as cell lines expressing each of the truncations and point mutants (Figure 5b). Western blot analysis of expression of the truncated and mutant CAIX proteins demonstrated that the proteins were expressed by the cells at similar levels, with slightly lower levels expressed by the mutant lacking the PG-like domain (Figure 5b). Furthermore, the CAIX variants localized at the cell surface (Supplementary Figure S5c), providing a panel of cell lines for use in interrogating the role of CAIX activity in regulating invasion and metastasis, both of which are dependent on collagen degradation.

Next, we employed this panel of cell lines to investigate the impact of the truncations and mutations on the enzymatic activity of CAIX when compared to WT CAIX. Previous reports have suggested that the IC domain of CAIX is required for the activity of the extracellular catalytic domain,^33^ but these studies did not measure the catalytic activity of CAIX directly. Here, we utilized an assay that assesses CAIX catalytic activity directly in live 4T1 cells by measuring the conversion of carbon dioxide (CO_2_) to bicarbonate (HCO_3_^−^) and protons (H^+^). To ensure that an equal amount of CAIX protein was used for each assay, experiments were carried with a defined cell number using the cell lines in which we had demonstrated equal levels of protein expression (Figure 5b). Depletion of mouse CAIX (shCAIX), or treatment of mouse CAIX depleted, WT human CAIX expressing cells (sh/WT) with U-104 results in substantial inhibition of CAIX activity (Figure 5c, Supplementary Figure S6a). Furthermore, deletion of the IC domain (ΔIC) also results in significant inhibition of activity (Figure 5c, Supplementary Figure S6a), whereas deletion of the PG domain (ΔPG), which has been reported previously to regulate CAIX activity in studies using purified proteins *in vitro* and not in cells,^34^ did not have a significant effect (Figure 5c, Supplementary Figure S6a). The T443A and S448A+Y449A point mutants also reduced CAIX catalytic activity to levels between the WT protein and the ΔIC mutant (Supplementary Figures S6b and c). It is important to note that the activity of CAIX is not required for the association with MMP14, since this association is maintained in the presence of U-104 (Supplementary Figure S6d).

Taken together, these data demonstrate that the IC domain is required for full catalytic activity of CAIX in cancer cells and raises the intriguing possibility that the IC domain may contribute to CAIX-mediated tumor cell invasion and metastasis by regulating CAIX catalytic activity.

Next, invasion assays using these cells were carried out in hypoxia to evaluate the functional contribution of CAIX in a biologically relevant environmental context. Silencing of endogenous CAIX expression resulted in a significant decrease in cell invasion through Matrigel-coated transwell filters, an effect that is rescued by expression of WT huCAIX (Figure 5d). Furthermore, deletion of either the IC or the PG domains of CAIX resulted in decreased invasion through Matrigel (Figure 5e), while expression of the S448A+Y449A mutant reduced invasion to the same level of that observed for cells expressing the ΔIC mutant (Supplementary Figure S6e). Although the T443A mutant also reduced invasion, the difference compared to WT CAIX was not statistically significant (Supplementary Figure S6e). In addition, treatment of 4T1 cells expressing WT huCAIX with U-104 also significantly reduced invasion through Matrigel (Figure 5f), implicating CAIX catalytic activity in the regulation of invasion.

The acidic extracellular microenvironment has been previously reported to enhance tumor cell invasion through the activation of proteases such as cathepsins and MMPs.^6, 35^ Since our findings indicated that CAIX and MMP14 associate and colocalize at functional invadopodia, and that CAIX contributes functionally to collagen degradation at these sites (see Figure 4), we assessed the role of CAIX and MMP14 on invasion through collagen. First, we determined whether depletion of CAIX regulated invasion through rat tail type I collagen, a known substrate of MMP14.^36^ Silencing of CAIX expression in hypoxia resulted in significant inhibition of invasion and this inhibition is rescued by expression of huCAIX (Supplementary Figure S6f). In addition, the ΔIC mutant, but not the ΔPG mutant, also showed significantly reduced invasion (Supplementary Figure S6g), suggesting that while both the IC and PG domains may regulate invasion through matrices rich in laminins such as Matrigel, the IC domain is selectively required for efficient invasion through type I collagen.

We next determined the impact of pharmacologic inhibition of CAIX catalytic activity or MMP14 function on invasion through collagen. Treatment of 4T1sh/WT cells with U-104 in hypoxia resulted in inhibition of invasion (Figure 5g), as did treatment with a specific, function blocking monoclonal anti-MMP14 antibody^37^ (Figure 5h). Furthermore, the combination of the two inhibitors further reduced invasion (Figure 5i), indicating that both CAIX and MMP14 contribute functionally to this invasive capability. These data suggest that CAIX potentially promotes MMP14-mediated invasion through type I collagen and raise the possibility that the CAIX-mediated extracellular acidification may regulate MMP14 function.

To determine whether the reduction in the capability to invade observed *in vitro* correlated with altered metastatic propensity *in vivo*, we carried out studies using models of spontaneous and experimental metastasis in the 4T1 system. The effects on invasion *in vitro* are mirrored by spontaneous lung metastases formation from orthotopic 4T1 tumors wherein the depletion of CAIX results in a dramatic reduction in lung metastases (Figure 5j) without significant changes in primary tumor weight (Supplementary Figure S6h). The inhibition of metastasis is reversed in the presence of WT CAIX (Figure 5j). Furthermore, we carried out experimental metastasis assays, an approach that examines the contribution of invasion during the extravasation and colonization steps of metastasis,^24^ using the panel of 4T1 cell lines expressing the truncated and point mutant variants of CAIX. Lung metastases formation after tail vein injection was significantly lower in 4T1shCAIX cells (Figure 5k), and was also much lower in cells expressing the IC- and PG-deleted mutants, relative to those expressing WT huCAIX (Figure 5k). Interestingly, the results of this assay showed that sh/WT cells rescued metastasis to levels slightly below those seen in the spontaneous metastasis assay (Figure 5j). The modest difference in the degree to which this cell line rescued the metastatic phenotype may be due to the fact that the spontaneous metastasis assay interrogates the full metastatic cascade, whereas the experimental metastasis studies focus on the later stages of metastasis. Importantly, however, expression of WT CAIX provided substantial rescue of the metastasis phenotype in the tail vein injection assay, restoring it to 80% of control levels (Figure 5k), suggesting the importance of CAIX in the invasive process during extravasation and colonization.

### CAIX associates with MMP14 through the intracellular domain of CAIX

**Figure 6:**
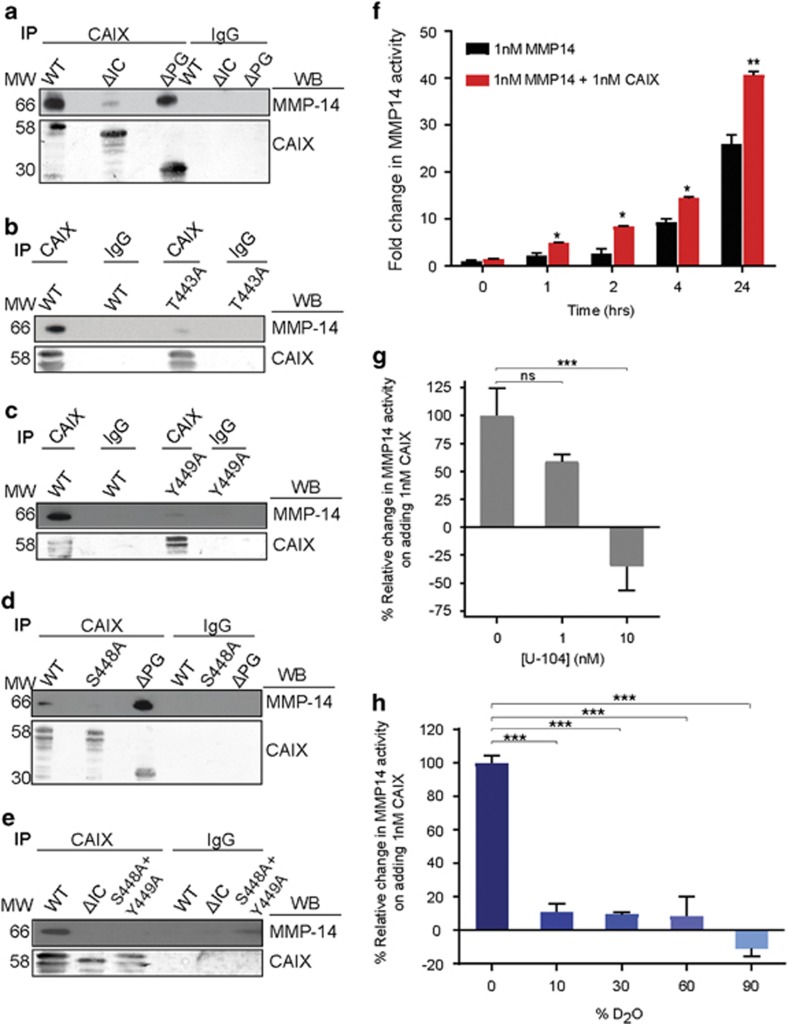
CAIX associates with MMP14 through its intracellular domain and potentiates MMP14 activity. (**a**) Co-IP of huCAIX and MMP14 from 4T1shCAIX cells cultured in hypoxia and expressing WT, ΔIC or ΔPG variants of huCAIX. (**b**) Co-IP of huCAIX and MMP14 from 4T1shCAIX cells cultured in hypoxia and expressing WT huCAIX or the T443A point mutant. (**c**) Co-IP of huCAIX and MMP14 from 4T1shCAIX cells cultured in hypoxia and expressing WT or the Y449A point mutant. (**d**) Co-IP of huCAIX and MMP14 from 4T1shCAIX cells grown in hypoxia and expressing WT huCAIX, the S448A point mutant or the ΔPG mutant. (**e**) Co-IP of huCAIX and MMP14 from 4T1shCAIX cells in hypoxia expressing WT huCAIX, the ΔIC truncation or the double point mutant S448A+Y449A. Isotype-specific IgG was used as a control for co-IPs. (**f**) Time-dependent stimulation of MMP14 activity in the presence (red bars) or absence (black bars) of equimolar concentrations of CAIX. Data show the mean±s.e.m. of technical replicates (*n*=5) and are representative of two independent experiments. *P<0.05, **P<0.01. (**g**) Dose-dependent inhibition of CAIX-mediated stimulation of MMP14 activity in the presence of increasing concentrations of U-104. Mean±s.e.m. of technical replicates (*n*=5). Data are representative of two independent experiments. ***P<0.001. (**h**) Inhibition of CAIX-mediated stimulation of MMP14 activity in the presence of increasing amounts of D_2_O (heavy water). Mean±s.e.m. of technical replicates (*n*=5). Data are representative of three independent experiments. ***P<0.001. Statistical analysis was performed using Student’s *t*-test (**f**) or ANOVA (**g**,**h**).

The association of CAIX with MMP14 is a particularly interesting finding since MMP14 is a cell surface metalloprotease involved in matrix degradation, tumor cell invasion and metastasis. To determine both the molecular basis of CAIX-mediated invasion and the critical domains of CAIX involved in this regulation, we utilized the 4T1 cells in which we had generated WT, truncation and point mutant variants of CAIX (see Figure 5a, Supplementary Figure S5a). We found that CAIX associates with MMP14 through the IC domain, since deletion of the IC domain, but not the PG domain, results in a significant decrease in co-IP of MMP14 relative to WT CAIX (Figure 6a, Supplementary Figure S7a).

Since our findings showed that the IC domain of CAIX is important for the association with MMP14, we wanted to identify regions within the IC domain that were potentially involved in mediating this novel association. Thr443 within the IC domain, a putative cyclic AMP-dependent protein kinase A (PKA) phosphorylation site, has been reported to regulate the extracellular activity of CAIX in conjunction with dephosphorylation of Ser448,^33^ suggesting that these closely aligned amino acid residues (Supplementary Figure S5a) may contribute to or regulate the association of CAIX with MMP14. While there is currently no known functional role for Y449, it was identified as a novel residue with potential involvement in the association as it is positioned immediately adjacent to S448 and is an additional putative phosphorylation site. Therefore, we analyzed the association, by co-IP, between CAIX and MMP14 in 4T1shCAIX cells cultured in hypoxia and expressing the point mutations T443A, S448A, Y449A and double point mutation, S448A+Y449A. Surprisingly, the association with MMP14 was substantially reduced in extracts from 4T1shCAIX cells expressing the T443A (Figure 6b, Supplementary Figure S7b) and Y449A (Figure 6c, Supplementary Figure S7c) mutations, relative to WT, and was strongly inhibited in extracts from the S448A (Figure 6d, Supplementary Figure S7d) and S448A+Y449A (Figure 6e, Supplementary Figures S7e) mutant expressing cells. These data suggest a critical role of these putative phospho-amino acid residues within the IC domain of CAIX in the association of CAIX with MMP14.

### CAIX stimulates MMP14 activity by providing hydrogen ions

It has previously been demonstrated that mammalian collagenases rely on H^+^ transfer for the cleavage of collagen.^38, 39^ It is well-known that CAIX activity acidifies pHe,^40^ and we have previously shown that depletion of CAIX expression by breast cancer cells inhibits acidification of pHe.^9^ Since our data demonstrate that CAIX colocalizes and associates with MMP14, and is required for MMP14-mediated collagen degradation and tumor cell invasion, we wanted to determine whether when colocalized, CAIX might stimulate MMP14-mediated collagen cleavage by locally providing H^+^ for the hydrogen transfer step in MMP14 catalysis. We co-incubated the catalytic domain of CAIX and the extracellular domain of MMP14 (including the collagen binding hemopexin domain) in the presence of type I DQ-collagen, and measured the release of fluorescence as the DQ collagen was degraded. The incubation of equimolar concentrations of MMP14 and CAIX increased MMP14 activity over time, compared to MMP14 alone (Figure 6f), which is itself active in the absence of CAIX (Supplementary Figure S8a). Furthermore, incubation of increasing amounts of CAIX dose-dependently augmented MMP14 activity (Supplementary Figure S8b). The CAIX-mediated increase in MMP14 activity is significantly and dose-dependently inhibited by the CAIX-specific inhibitor, U-104 (Figure 6g). Interestingly, the basal activity of MMP14 in this complex is reduced at high nanomolar concentrations. As a control, incubation of U-104 with MMP14 alone does not reduce its activity (Supplementary Figure S8c).

To try and understand the mechanism for this ‘activation’, we carried out the assay in deuterium oxide (D_2_O, aka heavy water) as described by Welgus *et al.*^39^ The stimulation of MMP14 activity by CAIX was abolished with increasing amount of D_2_O (Figure 6h), and was in fact inhibitory to the basal MMP14 activity at 90% D_2_O, similar to that observed with the CAIX inhibitor.

**Figure 7:**
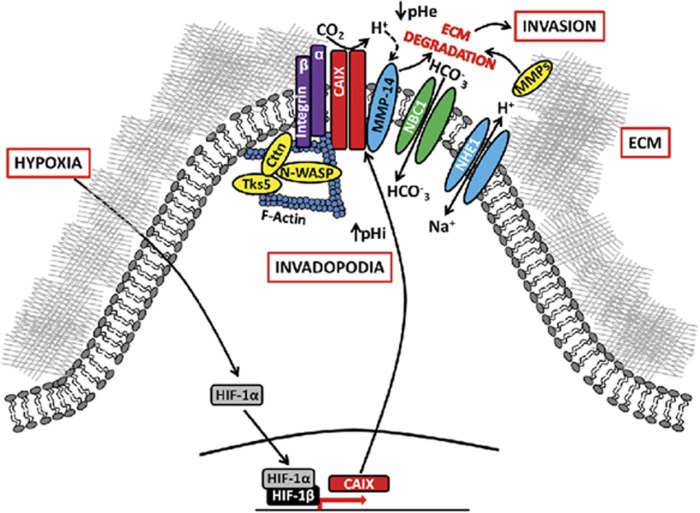
CAIX stimulates the activity of MMP14. Model for CAIX-mediated regulation of MMP14 activity. CAIX, which is upregulated in hypoxia, associates through its intracellular domain with MMP14 at functional invadopodia. At these invasive structures, CAIX functions to potentiate MMP14-mediated collagen degradation by directly contributing H^+^ ions required for MMP14 catalytic activity.

These data point to a novel mechanism of action for the CAIX-mediated stimulation of MMP14 activity whereby CAIX enhances MMP14 activity by increasing the rate of proton transfer required for MMP14 activity (Figure 7), and inhibition of CAIX activity negatively regulates MMP14 function.

## Discussion

There is considerable evidence that tumor hypoxia promotes both epithelial–mesenchymal transition and invasion,^41, 42^ and recent work has shown that cancer stem cells, which have undergone epithelial–mesenchymal transition, also reside in hypoxic niches.^11, 43^ However, the molecular basis of the connection between hypoxia and tumor invasion, especially the pathways involved in hypoxia-driven formation of invasive structures such as pseudopodia and invadopodia, which coalesce the matrix degradation machinery, is poorly understood. The role of CAIX, in particular, in hypoxia-mediated tumor cell invasion, or formation and/or function of invadopodia or pseudopodia has not been investigated to date. Here, we have demonstrated that CAIX is required for tumor cell invasion through matrigel as well as type 1 collagen. In addition, we have shown that both the PG and IC domains of CAIX have roles in invasion, and that the catalytic activity of CAIX is required since invasion is inhibited by a small molecule inhibitor of CAIX.

To identify potential CAIX-associating partners and investigate the mechanism of CAIX-mediated tumor cell invasion, we utilized a global, unbiased proteomic approach using a BioID analysis,^28^ a highly robust technique for mapping highly complex protein interactomes.^44^ The CAIX interactome that we identified reveals that CAIX associates with several metabolic cell surface transporters which may be critical for the metabolic response to hypoxia. More germane to the present focus of this study on tumor invasion, however, the BioID data also surprisingly demonstrate that CAIX associates with cell surface pro-invasive and matrix modeling proteins such as β1 integrins, integrin associated proteins (CD98hc) and MMP14. These observations are particularly intriguing given that previous studies using exogenously expressed MMP14 as bait in a melanoma cell line identified 158 potential proteins that associate with MMP14, including membrane proteins such as integrins, although these associations were not validated by co-IP.^45^ CAIX was not identified as an associating partner of MMP14 in this study, a result that is not surprising given that the experiments were performed only in normoxia, precluding expression and association of hypoxia-induced proteins.^45^

Interestingly, our analysis demonstrated that CAIX preferentially associates with collagen and laminin integrins. While these proteins can be clearly demonstrated to associate with CAIX by co-IP analysis, colocalization studies reveal that CAIX localizes preferentially to regions of cell motility and invasion, namely pseudopodia and invadopodia, known to be composed of integrins and MMP14. We have demonstrated here that CAIX colocalizes with the collagen receptor α2/β1 integrin and the collagen protease, MMP14 in pseudopodia-like protrusions that are positive for F-actin and cofilin, but not with F-actin or FAK at focal adhesions. It is noteworthy that while lamellipodia can be observed in some cancer cells, many protrusions of cancer cells do not correspond to the classic definition of lamellipodia and include both filopodia and blebbed spherical protrusions, thus making the use of the broader term, pseudopodia, relevant in this context.

While the data from the co-IP and colocalization experiments clearly validate the BioID data, it still needs to be determined whether the association of CAIX with integrins and MMP14 occur through direct protein–protein interactions. Importantly, however, the virtual elimination of the association of CAIX and MMP14 by co-IP upon deletion or mutation of the IC domain of CAIX (see Figure 6) suggests that this association occurs through direct protein–protein interactions. Detailed analysis of direct versus indirect interactions will be the subject of future in-depth biochemical studies.

In cancer cells, migration and invasion are highly integrated and dynamic processes. While our data showing the localization of CAIX to pseudopodia-like protrusions resembling lamellipodia point to a potential role of CAIX in cancer cell motility, our findings also extensively demonstrate that CAIX actively regulates invasion through migration-independent mechanisms that involve stimulation of degradation of collagen, likely through localized stimulation of MMP14 activity. Thus, we have demonstrated that CAIX is a component of mature invadopodia and colocalizes to these structures with markers of invadopodia.^21^ Furthermore, we have shown that inhibition of CAIX by genetic and pharmacologic strategies inhibits direct invasion through Matrigel and collagen, reduces collagen degradation, and that CAIX and MMP14 colocalize to functional invadopodia at regions of active matrix degradation as assessed using fluorescently labeled substrates. Together with our finding that the association of CAIX with MMP14 stimulates MMP14 activity (discussed below), our data suggest that, in addition to its effects on migration, CAIX has an important and as yet uncharacterized role in promoting cancer cell invasion.

The data presented here identify, for the first time, a role of CAIX in promoting tumor cell invasion through a strong association with MMP14, and regulating MMP14-mediated type I collagen-degradation activity. Our data suggest that the IC domain mediates the association with MMP14 and regulates the catalytic activity of CAIX, which is also required for CAIX-mediated invasion. Importantly, we demonstrate significant inhibition of CAIX function through the use of single domain truncation mutants of CAIX, relative to the WT protein, but residual functional activity remains evident. It is certainly possible that simultaneous disruption of multiple domains of CAIX may be necessary to achieve complete inhibition of function, especially given the complex CAIX interactome identified here using the BioID strategy. For example, our data demonstrate that both the IC domain and PG domain regulate invasion through Matrigel, a complex array of several extracellular matrix components, particularly basement membrane constituents such as laminins. In contrast, deletion of the IC domain, but not the PG domain, reduces both CAIX catalytic activity and invasion through collagen I, a major component of tumor stroma, suggesting that the domains of CAIX may demonstrate mechanistically distinct, yet complementary functions depending on the composition of the matrix. Thus, our data suggest that CAIX is functionally important both for invasion through the basement membrane and collagen I laden tumor stroma.

Our data also point to a critical role of a short region within the IC domain of CAIX in mediating its association with MMP14. This region contains three putative phosphorylation residues, the mutations of which dramatically reduce or abolish the association with MMP14, suggesting possible regulation between these two proteins through phosphorylation–dephosphorylation events. Alternatively, since serine and threonine residues can also be regulated through glycosylation,^46^ the regulation of the association and of invasion may be mediated by O-glycosylation.

To gain mechanistic insight into the role of CAIX in activation of MMP14, we analyzed MMP14 activity (collagen degradation) *in vitro* in the presence of CAIX. This cell-free analysis using purified proteins has demonstrated that CAIX is able to stimulate MMP14 activity *in vitro* in a manner that requires CAIX catalytic activity. Furthermore, by utilizing deuterium oxide,^39^ we have identified a potential mechanism for the stimulation of MMP14 activity by CAIX.

Our data suggest that CAIX, through its conversion of CO_2_ to bicarbonate and H^+^, provides protons critical for MMP14 activity. Thus, by associating strongly with MMP14, CAIX may further enhance MMP14 collagenolytic activity through stimulation of H^+^ transfer, a mechanism described several years ago for the regulation of mammalian MMPs.^38, 39^

Extracellular acidification has been postulated to activate proteases,^6^ and specifically, MMP14 showed increased type I collagen degradation activity *in vitro* at pH values<7.0.^47^ Along these lines, treatment of tumor-bearing mice with bicarbonate has been shown to decrease cathepsin and MMP activity within the tumors.^48^ Importantly, recent studies have linked expression of MMP14 with cancer progression and metastasis, and poor prognosis in patients.^49, 50, 51^ Significantly, a recent study has shown a strong inverse correlation between expression of MMP14 and metastasis-free survival of breast cancer patients, as well as an essential role of MMP14 in ductal carcinoma *in situ* transition to invasive carcinoma and in basement membrane invasion.^52^ This study also showed significant expression of MMP14 in subsets of triple-negative breast tumors, a finding that correlates with the expression of CAIX in over half of patients with triple-negative breast cancer.^9^ Furthermore, our findings provide functional and mechanistic surrogates for the recent demonstration that in stage 2 invasive breast cancers, CAIX is upregulated in the tumor edge consistent with an acid-producing invasive phenotype.^53^ Studies in gastric cancer have also shown that the presence of CAIX at the invasive front is associated with shortened post-operative survival and that restoration of CAIX expression by 5-azadeoxyctyidine-treated gastric cancer cell lines is associated with increased invasion.^54^ Our findings therefore suggest that the elevated co-expression of CAIX and MMP14 and their association could lead to local activation of MMP14 within invasive structures such as invadopoda, leading to the promotion of invasion and metastasis. It should be noted, however, that while the association of CAIX and MMP14 and CAIX-mediated effects MMP14 activity provide one mechanism for increased invasion and metastasis, CAIX may also potentiate metastasis by other mechanisms, for example by promoting the development of the pre-metastatic niche, as we have shown previously.^55^

In conclusion, we have uncovered novel functions of CAIX through a global proteomic approach, in regulating hypoxia-induced tumor cell invasion. Our data demonstrate, for the first time, that the association and colocalization of CAIX and MMP14 within mature invadopodia results in the promotion of MMP14-dependent matrix degradation, invasion and metastasis. Thus we provide mechanistic insights into hypoxia-driven invasion and identify a new protein complex for targeting tumor progression, especially since recent reports have demonstrated a critical role of invadopodia during the extravasation step of metastasis.^24^ Furthermore, CAIX-specific inhibitors are in clinical trials (www.Clinicaltrials.gov, ID # NCT02215850) and, therefore, our findings identify tumor invasion as a new target for these compounds.

## Materials and methods

### Reagents and antibodies

The CAIX antibodies (goat anti-mouse CAIX AF2344, goat anti-human CAIX AF2188, monoclonal mouse anti-human CAIX MAB2188) and the mouse anti-IgG_2A_ (MAB003) isotype control were obtained from R&D systems (Minneapolis, MN, USA). The mouse monoclonal anti-cortactin (ab33333), function-blocking mouse monoclonal anti-MMP14 (ab78738) and rabbit polyclonal anti-MMP14 (ab51074) antibodies were obtained from Abcam (Cambridge, MA, USA). The rabbit anti-Tks5 (aka FISH; sc-30122), goat anti-integrin β1 (M-106) (sc-6622), mouse anti-integrin β1 (P5D2) (sc-13590), goat anti-α2 integrin (N-19) (sc-6586), goat anti-CD98 (C-20) (sc-7095), mouse anti-CD98 (F-2) (sc-390154), and rabbit anti-FAK (A-17) (sc-557) antibodies were obtained from Santa Cruz Biotechnology (Santa Cruz, CA, USA). The mouse anti-β1-integrin (Cat no. 556048) antibody was obtained from BD Biosciences (Mississauga, Ontario, Canada). The mouse anti-α2-integrin antibody (clone-P1E6, Cat no. MAB 1950) was obtained from Millipore (Etobicoke, ON, Canada). The rabbit anti-cofilin (D3F9 XP®) (5175) antibody was obtained from Cell Signalling Technology Inc (Danvers, MA, USA). The mouse anti-β-actin antibody (A5441) was obtained from Sigma-Aldrich (St Louis, MO, USA).

### Cell culture

The 4T1 murine breast cancer cell line and MDA-MB-231 human breast cancer cell line were obtained from the American Type Culture Collection (ATCC). The MDA-231 LM2-4^luc+^ cells were provided by Dr Robert Kerbel (University of Toronto, Toronto, Canada). The PK-8 human pancreatic ductal adenocarcinoma cell line was provided by Drs Donald Yapp and Sylvia Ng (BC Cancer Research Centre, Vancouver, Canada).^56^ The MCF7 breast cancer cells, MCF10A mammary epithelial cells and HEK293 T-REx cells were obtained from ATCC and cultured as previously described.^57^ The MDA-MB-231 cell line was maintained in Dulbecco’s modified Eagle’s medium containing 25 mM glucose (Gibco cat # 11995-065, Burlington, Ontario, Canada) and supplemented with 10% fetal bovine serum (FBS; Gibco, Burlington, Ontario, Canada), while the 4T1 cell line also required the addition of non-essential amino acids (1 × NEAA). The PK-8 and MDA-MB-231 LM2-4^luc+^ cell lines were grown in RPMI-1640 medium containing 11 mM glucose (Gibco cat # 11835-030, Burlington, Ontario, Canada) supplemented with 10% FBS. For culture in normoxia, cells were incubated in a humidified incubator at 37 °C with 5% CO_2_. For studies in hypoxia, cells were grown at 37 °C in an atmosphere of 1% O_2_, 5% CO_2_, 94% N_2_ in a humidified incubator inside a sealed workstation as described previously.^11^ Cell lines were routinely tested for mycoplasma contamination using either the LookOut Mycoplasma PCR detection kit (Sigma-Aldrich, Oakville, ON, Canada; Cat. No. MP0035) or the MycoAlert Mycoplasma Detection kit (Lonza, Mississauga, ON, Canada). The MCF7, MCF10A and HEK293 T-REx cell lines have been authenticated using next generation RNAseq analyses. The remaining cell lines used in the manuscript have been authenticated using short tandem repeat DNA profiling (DNA fingerprinting) by a commercial testing facility (Genetica, Burlington, NC, USA). In addition, the cell lines were routinely tested for viability, morphology, hypoxia-induced endogenous CAIX expression or over-expression of CAIX and *in vivo* tumor growth.

### Cloning of CAIX truncation and point mutants

Full length WT human *CA9* cDNA cloned into the pTREX-A (pcDNA4/TO/myc-His A; Invitrogen Life Technologies, Burlington, ON, Canada) plasmid vector was a generous gift from Dr Jacques Pouyssėgur (University of Nice, France).^8, 9^ The construct encoding for the ΔIC mutant was generated by introduction of a stop codon after amino acid 433 by site-directed mutagenesis. The construct encoding for the ΔPG mutant was generated from two PCR products by amplification of the sequences corresponding to amino acids 1–37 and 130–459 using two sets of primers, followed by ligation and cloning of the product into the pcDNA4 vector. The CAIX intracellular domain single and double point mutants (T443A, S448A, Y449A and S448A+Y449A) were generated by site-directed mutagenesis. Plasmid DNAs were purified using a DNA purification kit (Qiagen, Toronto, ON, Canada) and constructs were sequenced to verify fidelity before transfection into cells.

### Cloning of CAIX-BirA* fusion protein

The human CAIX (WT) clone was amplified from the pcDNA4/TO/myc-His A vector. The BirA*-FLAG sequence was amplified from the pcDNA5 FRT/TO [MCS]‐BirA*‐FLAG vector. BirA* is an *Escherichia coli* biotin ligase with an Arg118Gly mutation as described previously.^58^ The CAIX-BirA* fusion protein was generated by an overlap extension PCR using a short Ala Ala Ala linker sequence linking the carboxy terminal of CAIX to the amino terminal domain of BirA*. The CAIX-BirA* construct was then cloned into the pENTR/SD/D-TOPO vector for the Gateway system using the pENTR Directional TOPO cloning kit (Cat no. K2420, Invitrogen Life Technologies). Furthermore, using the Gateway LR clonase II enzyme mix (cat no. 11791, Invitrogen Life Technologies), the CAIX-BirA* construct was placed into the gateway lentiviral destination vector-pLenti CMV Neo DEST (705-1) (Plasmid #17392, Addgene, Cambridge, MA, USA). For use as a negative control for BioID analysis, a FLAG-BirA* construct was also cloned into the final destination vector. Purified plasmids were sequenced before transduction into cells.

### Cas-9 mediated editing of *CA9*

The human *CA9* locus was disrupted by utilizing the pCRISPR-CG01 vector (Genecopoeia) co-expressing mCherry, WT *Cas9* from *Streptococcus pyogenes* and a sgRNA targeting exon 1. Targeted cells were transfected with Lipofectamine 2000 (Thermo Fisher Scientific, Waltham, MA, USA) as per the manufacturer’s instructions. After 72 h, FACS was utilized to select mCherry positive cells into single clones in 96-well plates. Colonies were screened by western blot for loss of CAIX expression. The *CA9* locus of positive clones was amplified using forward 5′-CTCCCCCACCCAGCTCTCG-3′ and reverse 5′-CCTGGATTTGGAGATTGATGACCAC-3′ primers and subjected to Sanger sequencing for confirmation and mutation analysis.

### Generation of stable cell lines

For stable depletion of human and mouse CAIX in MDA-MB-231 LM2-4^luc+^ and 4T1 cells, shRNAmir constructs (Open Biosystems, Huntsville, AL, USA) were transduced into cells using lentivirus as per the manufacturer’s instructions. Transduced cells were selected using puromycin. Stable 4T1shCAIX clones were derived by FACS of GFP-positive cells. Re-introduction of WT human (hu) CAIX or of the different constructs was accomplished by plasmid transfection into 4T1shCAIX cells using Lipofectamine 2000 as per the manufacturer’s instructions. Zeocin was used for selection of successfully transfected cells.

The MDA-MB-231-CAIX-BirA* cell line was generated using lentiviral transduction. The transduced cells were selected using G418. The cell line was tested for the expression of the CAIX-BirA* by western blot.

### BioID of cultured cells

Biotin labeling of cultured cells for BioID analysis was performed as described previously.^57^ Briefly, MDA-MB-231-CAIX-BirA* cells or MDA-MB-231-BirA* control cells were cultured in normoxia or hypoxia for 72 h and allowed to reach approximately 80% confluency. Cells were then incubated with media containing 50 μM biotin for 24 h to induce labeling. Cells were scraped into phosphate buffered saline (PBS), pooled, washed twice in 25 ml PBS, and collected by centrifugation at 1000 *g* for 5 min at 4 °C. Cell pellets were stored at −80 °C before downstream analysis.

### Biotin-streptavidin affinity purification for mass spectrometry

Cell pellets were resuspended in 10 ml of lysis buffer (50 mM Tris-HCl pH 7.5, 150 mM NaCl, 1 mM EDTA, 1 mM EGTA, 1% Triton X-100, 0.1% SDS, 1:500 protease inhibitor cocktail (Sigma-Aldrich), 1:1000 benzonase nuclease (Millipore) and incubated on an end-over-end rotator at 4 °C for 1 h, briefly sonicated to disrupt any visible aggregates, then centrifuged at 16 000 *g* for 30 min at 4 °C. Supernatant was transferred to a fresh 15 ml conical tube. Thirty microlitres of packed, pre-equilibrated Streptavidin sepharose beads (GE) were added and the mixture was incubated for 3 h at 4 °C with end-over-end rotation. Beads were pelleted by centrifugation at 2000 rpm for 2 min and transferred with 1 ml of lysis buffer to a fresh Eppendorf tube. Beads were washed once with 1 ml lysis buffer and twice with 1 ml of 50 mM ammonium bicarbonate (ammbic, pH 8.3). Beads were transferred in ammbic to a fresh centrifuge tube, and washed two more times with 1 ml ammbic buffer. Tryptic digestion was performed by incubating the beads with 1 μg MS-grade TPCK trypsin (Promega, Madison, WI, USA) in 200 μl of 50 mM ammonium bicarbonate (pH 8.3) overnight at 37 °C. The following morning, 0.5 μg MS-grade TPCK trypsin was added and the beads were incubated for two additional hours at 37 °C. Beads were pelleted by centrifugation at 2000 *g* for 2 min and the supernatant was then transferred to a fresh Eppendorf tube. Beads were washed twice with 150 μl of 50 mM ammbic and these washes were pooled with the first eluate. The sample was lyophilized and resuspended in buffer A (0.1% formic acid). One-fifth of the sample was analyzed per MS run.

### Mass spectrometry

High performance liquid chromatography was conducted using a pre-column (Acclaim PepMap 50 mm × 100 μm inner diameter (ID) pre-column) and Acclaim PepMap (500 mm × 75 μm diameter; C18; 2 μm;100 Å) Rapid Separation Liquid Chromatography (RSLC) column (Thermo Fisher Scientific), running a 120 min reversed-phase buffer gradient at 225 nl/min on a Proxeon EASY-nLC 1000 pump in-line with a Thermo Q-Exactive HF quadrupole-Orbitrap mass spectrometer. A parent ion scan was performed using a resolving power of 60 000 and then up to the 20 most intense peaks were selected for MS/MS (minimum ion count of 1000 for activation), using higher energy collision induced dissociation (HCD) fragmentation. Dynamic exclusion was activated such that MS/MS of the same *m/z* (within a range of 10 ppm; exclusion list size=500) detected twice within 5 s were excluded from analysis for 15 s. Data were analyzed using the trans-proteomic pipeline^59, 60^ via the ProHits software suite.^61^ For protein identification, Thermo.RAW files were converted to the.mzXML format using Proteowizard,^62^ then searched using X!Tandem^63^ and Comet^64^ against the human Human RefSeq Version 45 database (containing 36 113 entries). Search parameters specified a parent ion mass tolerance of 10 ppm, and an MS/MS fragment ion tolerance of 0.4 Da, with up to two missed cleavages allowed for trypsin. Variable modifications of +16@M and W, +32@M and W, +42@N-terminus, +1@N and Q were allowed. Proteins identified with a ProteinProphet confidence score of ⩾0.90 (corresponding to ⩽1% False Discovery Rate) and with ⩾2 unique peptides were analyzed with SAINT Express v.3.3. Two replicates of CAIX-BirA* were compared to four runs of BirA* alone expressed in MDA-MB-231 cells, and a set of 16 generic control runs (consisting of two no bait controls from MCF7 cells, 4 BirA* alone expressed in MCF10A cells, and 10 BirA* alone expressed in HEK293 T-REx cells). Control data were collapsed to the two highest spectral counts for each detected prey protein, and proteins with SAINT BFDR⩽0.01 are reported in Supplementary Table S1.

### Flow cytometry

1.0 × 10^6^ cells were trypsinized and re-suspended in FACS Buffer (PBS pH 7.4+3% FBS). Cells were incubated with either anti-mouse CAIX (R&D Systems, MAB2188) or a control anti-mouse IgG_2a_ (R&D Systems, MAB003) at 1:100 for 30 min on ice. Cells were washed with 1 ml FACS buffer and pelleted at 1200 rpm for 5 min. The supernatant was aspirated and cells were incubated with anti-mouse IgG_2A_ AlexaFluor 647 (Invitrogen Life Technologies, A21241) at 1:200 for 30 min on ice. In the case of MDA231 cells growing in hypoxia, the above two antibody incubation steps were performed in the hypoxia chamber. Cells were washed and resuspended in 200 μl of FACS buffer containing 1:10 000 propidium iodide (Sigma-Aldrich, P4170). 1.0 × 10^5^ events were collected for each sample on a BD FACS Calibur (BD Biosciences, San Jose, CA, USA) and the data were analyzed using FlowJo Software (FlowJo LLC, Ashland, OR, USA).

### Western blot analysis

Cells were grown in normoxia or hypoxia for 72 h to induce CAIX expression, followed by lysis at 4 °C in RIPA (50 mM Tris, pH 7.5, 150 mM NaCl, 1% NP-40, 0.1% SDS and 0.5% NaDoc) or NP-40 (10 mM Tris-HCl, pH 7.5, 150 mM NaCl and 1% NP-40) buffer. For treatment with CAIX inhibitor U-104, a final concentration of 50 μM was added to the media for the last 24 h in hypoxia. Equal amounts of lysate (15-30 μg) were loaded onto SDS–PAGE gels. Western blots were performed as described previously^65^ using goat anti-mouse CAIX (1:500), goat anti-human CAIX (1:500), rabbit anti-MMP14 (1:500), mouse anti- β1 integrin (1:400), goat anti- α2 integrin (1:200) and mouse anti-β-actin (1:10 000) primary antibodies.

### Co-immunoprecipitation

Cells were cultured in normoxia or hypoxia for at least 72 h and were lysed at 4 °C in NP-40 buffer containing protease inhibitor cocktail, 1 mM Na_3_VO_4_, 2 mM sodium fluoride and 1 mM phenylmethylsulfonyl fluoride. One to three milligrams of protein was immunoprecipitated at 4 °C overnight using either 30 μg of monoclonal anti-huCAIX antibody or 15 μg of monoclonal anti-MMP14 antibody covalently linked to CNBr-activated Sepharose 4B as described previously.^66^ In the case of co-IP with integrins, 1 mg of the cell lysate was immunoprecipitated at 4 °C overnight using 10 μg of monoclonal mouse anti- integrins β1and α2 and then incubated for 1 h at 4 °C with Protein A/G Agarose and UltraLink Resin (Cat no. 53132, ThermoFisher Scientific, Burlington, ON, Canada). The resin was extensively washed with NP-40 buffer pH 8.0 with 1% Tween-20, resuspended in sample buffer and boiled at 100 °C for 10 min under non-reducing conditions. The proteins in the supernatant were separated from the resin by centrifugation using a polypropylene spin column (Bio-Rad, Mississauga, Ontario, USA) for 1 min, at 4000 rpm. β-mercaptoethanol was added to the supernatant and eluates were boiled again. All samples were loaded on SDS–PAGE gels and western blots were performed as described above.

#### ‘In-cell’ co-IP

An ‘in-cell’ co-IP was performed to pull down CD98hc with CAIX. MDA-MB-231-CAIX-BirA* cells were cultured in normoxia at 1 × 10^4^ cells/cm^2^ for 72 h. Cells were detached using 0.5 mM EDTA in PBS, washed and incubated in serum-free Dulbecco’s modified Eagle’s medium supplemented with 2% (w/v) bovine serum albumin for 1 h at 4 °C with shaking. 3 × 10^6^ cells were incubated with 10 μg of mouse anti-huCAIX for 1 h at 4 °C. The cells were then lysed for 60 min at 4 °C in a mild lysis buffer (25 mM Tris-HCl, pH 7.5, 150 mM NaCl, 1 mM CaCl_2_ 1% Brij-35 with containing protease inhibitor cocktail, 1 mM Na_3_VO_4_, 2 mM sodium fluoride). The lysate was added to the Protein A/G beads for 1 h at 4 °C. The remainder of the co-IP procedure was performed as described above.

### Immunofluorescence

Acid washed glass coverslips were coated with 10 μg/ml of type 1 collagen (Cat no. A1048301, Life Technologies) or with 10 μg/ml of DQ collagen and incubated for 1 h at 37 °C and washed extensively with PBS. 2.5 × 10^4^ MDA-MB-231-CAIX-BirA* cells were plated onto the collagen-coated coverslips and incubated for 24 h in normoxia before staining, whereas 5 × 10^3^ WT MDA-MB-231 cells were plated onto collagen coated coverslips and incubated for 72 h in hypoxia before staining.

Since CAIX, MMP14, ITGB1 and ITGA2 are cell-surface antigens, staining for these proteins was performed before fixing the cells. Briefly, the cells were washed three times with ice cold Hanks buffered saline and then blocked with Hanks buffered saline+10% FBS on ice for 30 min. The coverslips were incubated with the primary antibodies for 30 min at 4 °C—goat anti-huCAIX (1:100), rabbit anti-MMP14 (1:500), mouse anti-β1 integrin (1:50), mouse anti- α2 integrin (1:50). The coverslips were washed three times with 1 × PBS and then fixed in 4% PFA for 10 min. FAK (Rabbit anti-FAK, 1:200) and cofilin (Rabbit anti-cofilin, 1:400) staining was performed on fixed cells that were permeabilized with 0.1% Triton X-100 for 10 min. The coverslips were then washed three times with PBS and incubated with AlexaFluor 488/594 conjugated secondary antibodies (1:400; Jackson ImmunoResearch Laboratories, West Grove, PA, USA) for 1 h at room temperature (RT). For F-actin staining, the permeabilized cells were incubated with Alexa 594 phalloidin (1:40, Cat no. A12381, Thermo Fisher scientific) for 10 min and washed with PBS. The coverslips were then mounted in Prolong Diamond anti-fade mounting media with 4’,6-diamidino-2-phenylindole (Cat no. P3697, ThermoFisher Scientific).

### Transfection of MDA-MB-231-CAIX-BirA* cells with GFP

2 × 10^4^ cells were seeded onto acid washed glass coverslips coated with 10 μg/ml of type 1collagen (Cat no. A1048301, Life Technologies) and were allowed to attach overnight. Five hundred nanograms of pcDNA3-*EGFP* (Addgene) was transiently transfected into these cells using 2.5 μl of lipofectamine 2000 (Thermo Fisher Scientific). GFP transfection was monitored using Incucyte ZOOM live cell analysis. Forty-eight hours after transfection, the cells were fixed and stained for CAIX, α2 integrin and MMP14.

### Immunofluorescence staining of invadopodia

Cells were plated onto collagen-coated coverslips as described above and the staining was performed on fixed and permeabilized cells by incubating with the primary antibodies for 1 h at RT—goat anti-moCAIX (1:100), goat anti-huCAIX (1:100), Rb anti-MMP14 (1:100), mouse anti-MMP14 (1:500), rabbit anti-Tks5 (1:50) and mouse anti-cortactin (1:400). The cells were then incubated with AlexaFluor 488/594/647 conjugated secondary antibodies for 1 h at RT.

### Imaging

Slides were imaged using either a Leica microscope equipped with a × 63 objective and a CCD camera or a Nikon Eclipse Ti microscope equipped with a × 60/1.49 NA objective and a CCD camera. The images were processed using ImageJ and Photoshop CS5 (v. 12.1). For imaging of cells transfected with GFP, 3D stack images were obtained using Intelligent Imaging Innovations (III) Zeiss spinning disk confocal microscope equipped with a 100 × /1.45 Zeiss oil Plan-Fluar objective at a 200 nm step size. For each sample, a 3D projection maximum was generated with Slidebook 6.0 (III).

### Preparation of collagen-coated tissue cultureware

For collagen degradation assays, 12-well plates were coated with rat tail type I collagen (Life Technologies) for 30 min at 37 °C, 5% CO_2_ in a humidified incubator as described for invasion assays. For invadopodia formation on gelatin, glass coverslips were coated with Oregon Green 488-gelatin (Life Technologies) or unlabelled gelatin as described previously.^67^ Briefly, coverslips were coated with 50 μg/ml poly-L-lysine followed by 0.5% gluteraldehyde, and inverted on a 80 μl drop of gelatin for 10 min. Coated coverslips were then incubated with 5 mg/ml NaBH_4_ and washed extensively with PBS. For invadopodia formation on DQ collagen, glass coverslips were coated with 400 μl of 10 μg/ml DQ Collagen, type I (Life technologies), incubated for 1 h at 37 °C and washed extensively with PBS.

### Cell-based DQ collagen degradation assay

A 96-well tissue culture blk/clr plate was coated with 100 μl of a 1:10 mixture of 0.1 mg/ml DQ collagen, Type I (Cat no. D12060, Thermo Fisher Scientific) and 0.1 mg/ml rat tail collagen, Type I (A1048301, Thermo Fisher Scientific), incubated at 37 °C in 5% CO_2_ incubator for 1 h and washed three times with PBS. 3 × 10^4^ cells (with or without U-104 treatment) were plated per well and incubated for 24 h at 37 °C in a 5% CO_2_ incubator. The fluorescence data were acquired on a SpectraMax i3 plate reader (Molecular Devices, Sunnyvale, CA, USA) at an excitation wavelength of 485 nm and an emission wavelength of 535 nm. The number of cells in each well was measured by staining the wells with 0.1% crystal violet for 1 h. After extensive washing with water, the crystal violet was dissolved in 10% acetic acid solution and absorbance values were read at 570 nm. Finally, the fluorescence values were normalized by dividing the fluorescence by the crystal violet absorbance values in each well. Data were analyzed using Graphpad Prism (La Jolla, CA, USA).

### Invadopodia formation and imaging

#### Gelatin

5 × 10^4^ cells were seeded on the gelatin-coated coverslips and incubated for 6 h in hypoxia, after which they were fixed in ice cold methanol (MeOH) for 10 min. For treatment with U-104, cell suspensions were pre-incubated in the presence of the inhibitor (50 μM) for 30 min at RT on a rotator prior to being seeded on gelatin in the presence of U-104.

#### DQ collagen

2.5 × 10^4^ MDA-MB-231-CAIX-BirA* cells were plated onto these coverslips overnight. The cells were fixed in 4% PFA for 10 min and immunostained.

### Chemotaxis migration assay

Chemotactic cell migration was performed using the IncuCyte ClearView 96 well Cell Migration Plate (cat no. 4582, Essen Biosciences, Ann Arbor, MI, USA). The design of the plate is based on the transwell Boyden chambers where each well is composed of a membrane insert (with 8 μm pores) and a reservoir. Indicated cell lines were serum starved for 24 h, trypsinized and 2 × 10^3^ cells were added to the upper chamber of each well. epidermal growth factor (10 ng/ml) was added as a chemo-attractant to the lower reservoir. The migration was monitored for 24 h within the incubator at 37 °C, 5% CO_2_ and analyzed using the Incucyte ZOOM live cell analysis instrument. Images were taken every 2 h with a Nikon Plan Fluor × 10/0.3 NA objective in the bright field mode. Migration was calculated as the total area occupied by the cells that migrated to the lower side of the membrane. This value was normalized to the total area of the cells occupied at *t*=0 h on the upper membrane.

### Wound-induced migration assay

The scratch wound migration assay was performed using the Incucyte ZOOM live cell analysis instrument (Essen Biosciences). To form a monolayer of the cells, the indicated cell lines were plated (3 × 10^4^ cells/ well) in 96-well Imagelock plates (Cat no. 4379, Essen Biosciences) 18 h before the start of the assay. To inhibit cell proliferation during the course of the assay, the cells were treated with 5 μg/ml Mitomycin C (Cat no. M4287, Sigma-Aldrich) for 2 h. A 96-pin wound-making tool (Woundmaker) was used to create a uniform wound in every well. The wound area of the cells growing in normoxia were imaged every 2 h for a total of 24 h with a Nikon Plan Fluor × 10/0.3 NA objective in the bright field mode. Cells growing in the hypoxia were imaged only at the zero and the 24 h time points. Migration was calculated as the percentage of the wound area occupied by the cells over time and values were normalized to the average value for the dimethylsulphoxide control (0 μM U-104). Values are expressed as the percentage difference compared to the dimethylsulphoxide control.

### Invasion assays

Cells were cultured for 72 h in hypoxia to induce CAIX expression, followed by serum-starvation for 24 h in hypoxia. Cells were then trypsinized, counted and 5 × 10^4^ or 1 × 10^5^ cells were seeded onto either 8 μm transwells coated with Matrigel (BD Biosciences) or 3 μm transwells (Corning, Durham, NC, USA) coated with rat tail type I collagen (Life Technologies, 2.25 mg/ml collagen; pH adjusted with NaOH and acetic acid as per the manufacturer’s instructions). Media supplemented with 10% FBS and 100 ng/ml rEGF (Sigma-Aldrich) was used as a chemoattractant. For treatment with U-104 (50 μM) or function-blocking anti-MMP14 antibody (20 μg/ml), cell suspensions were pre-incubated for 30 min at RT with rotation, followed immediately by transfer onto the transwell filters. Cells were allowed to invade for 24 h in hypoxia after which non-invasive cells were removed from the top chamber with a cotton swab. Membranes were fixed for 10 min with ice cold MeOH and the nuclei of invasive cells were stained with Hoescht 33342 (2 μg/ml) for 10 min (RT). The number of invasive cells was obtained from at least five different fields at low magnification (× 10) by using a Zeiss Observer.Z1 microscope and a CCD camera. For experiments using genetic depletion or pharmacologic inhibition of CAIX, data were normalized to the average value for the shNS or dimethylsulphoxide control samples as appropriate.

### Apoptosis assay

TUNEL (Terminal deoxynucleotidyl transferase dUTP nick end labeling) was performed according to the manufacturer’s instructions. Briefly, cells were incubated for 72 h in hypoxia prior to culture on coverslips for 24 h in hypoxia and low serum conditions (0.1% FBS). Cells were fixed and stained with the TUNEL reaction (Roche Diagnostics, Basel, Switzerland). Random fields were imaged using a Leica microscope equipped with a × 20 objective and a CCD camera. Image analysis was performed using ImageJ (v1.45s, National Institutes of Health, Bethesda, MD, USA).

### Mouse tumor models

All animal studies and procedures were performed in accordance with protocols approved by the Institutional Animal Care Committee at the BC Cancer Research Centre and University of British Columbia (Vancouver, BC, Canada). 4T1 tumor cell lines were inoculated into 7–9-week-old female BALB/c mice (Simonsen Laboratories, Gilroy, CA, USA) as described previously.^9^ For experiments involving spontaneous metastasis, 1 × 10^6^ cells/animal were implanted orthotopically into the mammary fat pad and tissues were collected at day 26 post-tumor cell inoculation. For studies involving experimental metastasis, 5 × 10^5^ cells/animal were injected through the tail vein and tissues were collected at 16 days post-injection (experimental end point for 4T1shNS group). Mice were not randomized but were instead allocated to different groups based on cell lines injected. The investigators were not blinded. The sample size estimate (*n*=6) was chosen based on previous studies that demonstrated an *n*=6 was adequately powered to detect a significant change between groups. No mice were excluded during the course of these studies.

### Clonogenic assays

Cells were dissociated from collected lung tissue by enzymatic digestion as described previously.^68^ Erythrocytes were lysed using ACK buffer (155 mM NH_4_Cl, 10 mM KHCO_3_ and 240 μM EDTA), cells were enumerated and aliquots of 2.5 × 10^3^ to 1 × 10^6^ cells were plated in media containing 60 μM 6-thioguanine (Sigma-Aldrich) to allow the exclusive growth of 4T1 cells. Cells were incubated for 10 days (37 °C, 5% CO_2_) before staining colonies with malachite green for quantification. The total number of clonogenic tumor cells in the lungs was calculated by multiplying the proportion of colony-forming tumor cells by the total number of cells recovered from the lungs.

### CAIX ‘in-cell’ activity measurement

The catalytic activity of CAIX was assessed by a modification of methods previously described.^69^ Briefly, cells were trypsinized, counted and resuspended in cold CO_2_-free isotonic buffer (20 mM Hepes, pH 8.0, 130 mM NaCl, 5 mM KCl). 0.8 ml of cell suspension (5 × 10^5^ cells) was added to a 4 ml borosilicate tube and equipped with a magnetic stir bar and a narrow pH electrode to monitor the change in pH. The reaction was performed on ice and was initiated by addition of 0.2 ml of CO_2_-saturated water. To determine the rate of spontaneous CO_2_ hydration, measurements were performed on cell-free (‘buffer’) samples. The increase in hydration rate above the spontaneous rate is a measure of carbonic anhydrase (CA) activity. Background CA activity in 4T1 cells was determined using 4T1shCAIX cell preparations. Inhibition of CAIX activity was assessed after 30 min incubation of cells with U-104 (50 μM) at 4 °C.

The time required for the buffer pH to drop from initial pH to the pH value half-way between the maximum and minimum pH was analyzed and defined as minimum activity (0%). 100% activity was defined as the time, in seconds, required to achieve the same drop in pH in the presence of WT huCAIX expressing cells alone. The percent activity was plotted against time and the equation derived from this standard curve was used to calculate percent inhibition in the presence of the CAIX mutants, based on the time required for the pH of in these reactions to drop from 8.0 to the pH value half-way between the maximum and minimum pH.

### *In vitro* MMP14 activity assay with DQ collagen, type I

20 mM HEPES pH 8.0, 150 mM NaCl, 5 mM CaCl_2_, 5 μM ZnCl_2_ was used as the assay buffer. Indicated concentrations of recombinant CAIX (Cat no. 2188-CA, R&D systems) were pre-incubated with activated MMP14 (Recombinant pro-MMP14, Cat.no. 918-MPN, R&D systems) for 30 min at RT. DQ Collagen (37.5 μg/1 ml reaction) was then added to the CAIX-MMP14 complex and incubated for 24 h at 37 °C, 5% CO_2_. The fluorescence intensity was measured at *λ*_excitation_ 485 nm and *λ*_emission_ 535 nm. For the CAIX inhibitor experiments, 40 μM stock of U-104 was diluted to the indicated concentrations in the assay buffer and pre-incubated with MMP14 or CAIX for 30 min at RT. The CAIX/U-104 mixtures were then incubated with MMP14 for a further 30 min at RT, DQ Collagen was added and assays were performed as described above. The effect of deuterium oxide (D_2_O, aka heavy water) on the activity of 1 nM MMP14 and that of an equimolar mixture of 1 nM MMP14 with 1 nM CAIX was determined in the assay buffer made in increasing concentrations of D_2_O (Cat no. 151882, Sigma-Aldrich).

### Statistical analysis

Statistical analyses were performed using GraphPad Prism 7. For comparison of two data sets containing three or more replicates, a two-tailed, unpaired Student’s *t*-test with Welch’s correction for unequal variances was performed. For instances in which greater than two data sets were compared, a one-way ANOVA was performed and Dunnet’s test was used to correct of multiple comparisons. Statistical significance was set at *P*<0.05.

## Figures and Tables

**Table 1:** Selected CAIX-BirA*FLAG high-confidence proximal associating proteins identified by BioID in MDA-MB-231 cells

*Functional class*	*Gene symbol*	*Full name*	*Description*	*Controls*		*Pool A*	*Pool B*	*SAINT score*
Transporters	*SLC3A2*	Solute carrier family 3 member 2	Amino acid transport heavy chain subunit CD98hc	37	35	116	115	1.00
	*SLC38A2*	Solute carrier family 38 member 2	Glutamine transporter SNAT2	12	12	56	61	1.00
	*SLC4A7*	Solute carrier family 4 member 7	Sodium bicarbonate transporter NBC2/3	5	4	52	28	1.00
	*SLC27A4*	Solute carrier family 27 member 4	Fatty acid transporter FATP4	6	5	18	23	0.99
Cell adhesion	*ITGB1*	Integrin subunit β1		11	9	66	45	1.00
	*ITGA6*	Integrin subunit α6		7	5	54	48	1.00
	*ITGA2*	Integrin subunit α2				51	47	1.00
	*ITGA3*	Integrin subunit α3		4		46	24	1.00
	*ITGA5*	Integrin subunit α5				9	6	1.00
Matrix remodeling	*MMP14*	Matrix metallopeptidase 14		5	3	18	31	1.00

For each associating protein, the ‘controls’ column shows the top two spectral counts detected between the 20 control runs. Pool A and B columns display the peptide counts detected in each of the biological replicates, and their corresponding SAINT score is shown in the adjacent column.
