# Supplementary material for: The interactome of metabolic enzyme carbonic anhydrase IX reveals novel roles in tumor cell migration and invadopodia/MMP14-mediated invasion
Source: Oncogene. 2017 Jul 10;36(45):6244–61. doi: 10.1038/onc.2017.219 (PMC5684442; doi:10.1038/onc.2017.219)
Supplement: Supplementary Table S1 [file onc2017219x1.pdf]

Supplemental Table

Table S1: BioID Mass spectrometry analysis. BioID results for: twenty control mass spectrometry (MS) runs (only the two spectral counts for each protein ID are displayed, see Method section for details), and two biological replicates of CAIX-BirA*Flag overexrpressing MDA-MB-231 cells cultured in normoxia (in black text) and in hypoxia (in blue text). Data are presented as spectral counts detected for each prey protein, as indicated. High confidence proximal interactors were determined with the SAINT algorithm, and filtered with the following parameters: iProphet confidence score p≥0.9, BFDR<0.02. Bait peptide counts (for BirA* and CAIX proteins) are highlighted in yellow (NA: Not Applicable).

						CA9 BioID normoxia			CA9 BioID hypoxia		
**Gene ID**	**Protein ID**	**Gene Name**	**Full name**	**Top 2 controls**		**pool A**	**pool B**	**SAINT**	**pool A**	**pool B**	**SAINT**
948469	16131807	**birA**	*biotin ligase (E.Coli)*	12451	11921	4760	2317	NA	4831	2565	NA
768	169636420	**CA9**	*carbonic anhydrase 9*			1048	661	NA	1062	620	NA
7112	73760405	**TMPO**	*thymopoietin*	108	108	1363	482	1.00	1290	397	1.00
6184	4506675	**RPN1**	*ribophorin I*	71	65	301	291	1.00	313	257	1.00
10424	291621647	**PGRMC2**	*progesterone receptor membrane component 2*	41	37	250	253	1.00	262	235	1.00
10970	19920317	**CKAP4**	*cytoskeleton-associated protein 4*	54	51	208	189	1.00	228	194	1.00
201595	30578410	**STT3B**	*STT3B, catalytic subunit of the oligosaccharyltransferase complex*	8	6	135	114	1.00	124	106	1.00
6520	61744483	**SLC3A2**	*solute carrier family 3 member 2*	37	35	116	115	1.00	110	73	
7444	194440707	**VRK2**	*vaccinia related kinase 2*	7	7	108	95	1.00	118	114	1.00
3163	187761307	**HMOX2**	*heme oxygenase 2*	5	5	111	86	1.00	122	93	1.00
7466	224994203	**WFS1**	*wolframin ER transmembrane glycoprotein*	6	3	108	85	1.00	124	74	1.00
5770	4506289	**PTPN1**	*protein tyrosine phosphatase, non-receptor type 1*	25	23	107	84	1.00	95	69	1.00
6646	49533617	**SOAT1**	*sterol O-acyltransferase 1*	6		111	80	1.00	108	57	1.00
3998	5031873	**LMAN1**	*lectin, mannose binding 1*	4	3	103	65	1.00	96	62	1.00
6734	23308697	**SRPR**	*SRP receptor alpha subunit*	13	12	100	53	1.00	102	45	1.00
55129	94536803	**ANO10**	*anoctamin 10*	4		82	60	1.00	88	55	1.00
84803	21362092	**AGPAT9**	*lysophosphatidylcholine acyltransferase 1*	11	8	77	52	1.00	72	46	1.00
6786	21070997	**STIM1**	*stromal interaction molecule 1*	12	9	67	60	1.00	76	48	1.00
10550	5453704	**ARL6IP5**	*ADP ribosylation factor like GTPase 6 interacting protein 5*	3	2	65	59	1.00	67	60	1.00
55591	155030244	**VEZT**	*vezatin, adherens junctions transmembrane protein*	3	2	68	55	1.00	76	42	1.00

81502	23308607	**HM13**	*histocompatibility (minor) 13*	6	4	57	62	1.00	47	48	1.00
84061	215983058	**MAGT1**	*magnesium transporter 1*			55	63	1.00	55	58	1.00
54407	21361602	**SLC38A2**	*solute carrier family 38 member 2*	12	12	56	61	1.00	57	62	1.00
23592	7706607	**LEMD3**	*LEM domain containing 3*	16	16	51	64	1.00	51	49	1.00
9694	7661910	**TTC35**	*ER membrane protein complex subunit 2*	9	7	62	50	1.00	60	33	1.00
3688	19743813	**ITGB1**	*integrin subunit beta 1*	11	9	66	45	1.00	72	38	1.00
57003	171906582	**CCDC47**	*coiled-coil domain containing 47*	9	8	61	46	1.00	62	40	1.00
3655	119395740	**ITGA6**	*integrin subunit alpha 6*	7	5	54	48	1.00	56	40	1.00
81839	20373171	**VANGL1**	*VANGL planar cell polarity protein 1*	5	4	61	41	1.00	64	40	1.00
3673	116295258	**ITGA2**	*integrin subunit alpha 2*			51	47	1.00	43	36	1.00
81542	151101292	**TMX1**	*thioredoxin related transmembrane protein 1*	5	3	57	41	1.00	56	30	1.00
55706	271398350	**TMEM48**	*NDC1 transmembrane nucleoporin*	8	8	57	36	1.00	54	27	1.00
51635	7706318	**DHRS7**	*dehydrogenase/reductase (SDR family) member 7*			56	36	1.00	53	33	1.00
493856	56605994	**CISD2**	*CDGSH iron sulfur domain 2*	13	13	45	38	1.00	45	29	
26092	389886539	**TOR1AIP1**	*torsin 1A interacting protein 1*	7	7	51	32	1.00	62	25	1.00
9497	385648268	**SLC4A7**	*solute carrier family 4 member 7*	5	4	52	28	1.00	32	24	1.00
23065	22095331	**KIAA0090**	*ER membrane protein complex subunit 1*	7	7	53	25	1.00	56	24	1.00
147007	22748979	**TMEM199**	*transmembrane protein 199*			42	32	1.00	36	48	1.00
64215	21361912	**DNAJC1**	*DnaJ heat shock protein family (Hsp40) member C1*			44	28	1.00	40	26	1.00
3675	4504747	**ITGA3**	*integrin subunit alpha 3*	4		46	24	1.00	47	29	1.00
163590	21450775	**TOR1AIP2**	*torsin 1A interacting protein 2*	9	9	39	31	1.00	48	26	0.96
5771	333108231	**PTPN2**	*protein tyrosine phosphatase, non-receptor type 2*	8	8	34	34	1.00	31	27	1.00
537	17136148	**ATP6AP1**	*ATPase H+ transporting accessory protein 1*			43	23	1.00	42	29	1.00
4162	71274107	**MCAM**	*melanoma cell adhesion molecule*	4	3	48	17	1.00	46	18	1.00
23190	24307965	**UBXN4**	*UBX domain protein 4*	8	5	37	27	1.00	45	31	1.00
23478	7657609	**SEC11A**	*SEC11 homolog A, signal peptidase complex subunit*	5	5	37	24	1.00	35	20	1.00
9789	162417971	**SPCS2**	*signal peptidase complex subunit 2*	2		32	28	1.00	28	22	1.00
23353	195972890	**SUN1**	*Sad1 and UNC84 domain containing 1*			30	30	1.00	32	29	1.00
9451	134304838	**EIF2AK3**	*eukaryotic translation initiation factor 2 alpha kinase 3*			32	23	1.00	30	32	1.00

203547	63025214	**VMA21**	*VMA21 vacuolar H+-ATPase homolog (S. cerevisiae)*			32	21	1.00	30		
23155	115270970	**CLCC1**	*chloride channel CLIC like 1*	4	4	34	16	1.00	32	22	1.00
3107	339882741	**HLA-C**	*major histocompatibility complex, class I, C*	5	3	22	28	1.00	23	26	1.00
60559	11345462	**SPCS3**	*signal peptidase complex subunit 3*	6	5	23	27	1.00	24	26	1.00
4323	4826834	**MMP14**	*matrix metallopeptidase 14*	5	3	18	31	1.00	15	24	0.99
65983	226443007	**GRAMD3**	*GRAM domain containing 3*	3	3	22	26	1.00	21	18	1.00
550	31712030	**AUP1**	*ancient ubiquitous protein 1*	2		25	22	1.00	21	19	1.00
29956	31077094	**CERS2**	*ceramide synthase 2*	3	2	24	23	1.00	25	15	1.00
6745	169404009	**SSR1**	*signal sequence receptor, alpha*	6	2	23	21	1.00	21	25	1.00
3482	119964726	**IGF2R**	*insulin like growth factor 2 receptor*	5	5	18	25	1.00	21	19	1.00
25923	45827806	**ATL3**	*atlastin GTPase 3*	3		24	18	1.00	16	15	0.99
10159	15011918	**ATP6AP2**	*ATPase H+ transporting accessory protein 2*			28	14	1.00	25	16	1.00
9296	20357547	**ATP6V1F**	*ATPase H+ transporting V1 subunit F*	2		25	17	1.00	25	27	1.00
128869	17998700	**PIGU**	*phosphatidylinositol glycan anchor biosynthesis class U*	4	3	24	18	1.00	28	15	1.00
10999	40807357	**SLC27A4**	*solute carrier family 27 member 4*	6	5	18	23	0.99	20	14	
51368	193788629	**TEX264**	*testis expressed 264*	2		21	18	1.00	25	15	1.00
752	33356148	**FMNL1**	*formin like 1*	7	4	22	17	0.96	13	13	
51170	142976729	**HSD17B11**	*hydroxysteroid (17-beta) dehydrogenase 11*			13	24	1.00	11	9	1.00
64755	257900512	**C16orf58**	*chromosome 16 open reading frame 58*			19	17	1.00	26	22	1.00
57326	19923830	**PBXIP1**	*pre-B-cell leukemia homeobox interacting protein 1*			15	21	1.00	19	14	1.00
196527	218156299	**ANO6**	*anoctamin 6*			21	14	1.00	23	13	1.00
3949	307775412	**LDLR**	*low density lipoprotein receptor*			12	22	1.00	10	24	0.95
9645	7662284	**MICAL2**	*microtubule associated monooxygenase, calponin and LIM domain containing 2*	2		20	12	1.00	15	15	1.00
57181	187936949	**SLC39A10**	*solute carrier family 39 member 10*			15	17	1.00	14	19	1.00
25777	313760643	**SUN2**	*Sad1 and UNC84 domain containing 2*			16	16	1.00	26	30	1.00
9854	42716277	**C2CD2L**	*C2CD2 like*			25	6	1.00	23	11	1.00
57620	281182822	**STIM2**	*stromal interaction molecule 2*	4	3	15	16	1.00	17	14	1.00
1047	194578888	**CLGN**	*calmegin*	4		18	13	0.93	21	14	0.96
9791	7662647	**PTDSS1**	*phosphatidylserine synthase 1*	2	2	21	9	0.98	25	21	1.00

202018	130977756	**TAPT1**	*transmembrane anterior posterior transformation 1*			16	13	1.00	13	8	1.00
55219	31542661	**TMEM57**	*transmembrane protein 57*	3	2	18	11	0.99	13	19	1.00
121457	24233517	**IKBIP**	*IKBKB interacting protein*			13	13	1.00	13	8	1.00
24145	39995064	**PANX1**	*pannexin 1*			18	8	1.00	20	20	1.00
10493	18379349	**VAT1**	*vesicle amine transport 1*	2		17	9	0.98	13	12	1.00
26090	109689718	**ABHD12**	*abhydrolase domain containing 12*			14	10	1.00	17	9	1.00
23341	56687498	**DNAJC16**	*DnaJ heat shock protein family (Hsp40) member C16*			15	9	1.00	16	13	1.00
351	209862833	**APP**	*amyloid beta precursor protein*			10	13	1.00	13	12	1.00
23545	42741679	**ATP6V0A2**	*ATPase H+ transporting V0 subunit a2*			10	13	1.00	9	13	1.00
9415	4758334	**FADS2**	*fatty acid desaturase 2*			8	15	1.00	6	9	1.00
9197	300360496	**SLC33A1**	*solute carrier family 33 member 1*	3	2	13	10	0.98	14	10	0.98
5447	127139033	**POR**	*cytochrome p450 oxidoreductase*			16	6	1.00	16	5	1.00
81671	20070349	**VMP1**	*vacuole membrane protein 1*			12	10	1.00	14	10	1.00
28972	193290128	**SPCS1**	*signal peptidase complex subunit 1*			12	9	1.00	11	8	1.00
137964	30520329	**AGPAT6**	*glycerol-3-phosphate acyltransferase 4*			10	10	1.00	14	16	1.00
811	4757900	**CALR**	*calreticulin*			6	14	1.00	3	8	0.99
6890	9665248	**TAP1**	*transporter 1, ATP-binding cassette, sub-family B (MDR/TAP)*			9	11	1.00	14	12	1.00
6526	110835708	**SLC5A3**	*solute carrier family 5 member 3*			8	11	1.00	10	6	1.00
7078	4507513	**TIMP3**	*TIMP metallopeptidase inhibitor 3*			9	9	1.00	6		
8677	4507285	**STX10**	*syntaxin 10*			8	9	1.00	10		
55151	8922461	**TMEM38B**	*transmembrane protein 38B*			13	4	1.00	9	4	1.00
118429	224809466	**ANTXR2**	*anthrax toxin receptor 2*			14	3	0.99	11	8	1.00
54587	14150145	**MXRA8**	*matrix-remodelling associated 8*			15	2	0.97	21		
22908	190014578	**SACM1L**	*SAC1 suppressor of actin mutations 1-like (yeast)*	2		8	9	0.96	3		
3106	17986001	**HLA-B**	*major histocompatibility complex, class I, B*	2	2	8	9	0.93	8	9	0.93
161176	145580592	**C14orf49**	*spectrin repeat containing, nuclear envelope family member 3*			7	9	1.00	4	14	1.00
7991	30410790	**TUSC3**	*tumor suppressor candidate 3*			5	11	1.00			
25966	119226222	**C2CD2**	*C2 calcium-dependent domain containing 2*	2		8	8	0.94	3		
79188	13236587	**TMEM43**	*transmembrane protein 43*	2		8	8	0.94	10	12	0.99

481	4502277	**ATP1B1**	*ATPase Na+/K+ transporting subunit beta 1*	8	7	1.00	9	8	1.00
11118	308736964	**BTN3A2**	*butyrophilin subfamily 3 member A2*	5	10	1.00	5	7	1.00
3678	56237029	**ITGA5**	*integrin subunit alpha 5*	9	6	1.00	12		
80856	38176151	**KIAA1715**	*KIAA1715*	10	5	1.00	13	6	1.00
2030	118582260	**SLC29A1**	*solute carrier family 29 member 1 (Augustine blood group)*	11	4	1.00	12	7	1.00
8829	182508169	**NRP1**	*neuropilin 1*	3	12	0.99		3	
1509	4503143	**CTSD**	*cathepsin D*	3	11	0.99	2		
54822	148612863	**TRPM7**	*transient receptor potential cation channel subfamily M member 7*	8	5	1.00	4	8	1.00
55850	8923936	**USE1**	*unconventional SNARE in the ER 1*	9	4	1.00	8	9	1.00
129642	40548387	**MBOAT2**	*membrane bound O-acyltransferase domain containing 2*	7	5	1.00	8		
768211	146094506	**RELL1**	*RELT like 1*	4	8	1.00	7	7	1.00
148867	222080086	**SLC30A7**	*solute carrier family 30 member 7*	7	5	1.00	8		
10094	5031597	**ARPC3**	*actin related protein 2/3 complex subunit 3*	2	10	0.97		7	
64866	30410805	**CDCP1**	*CUB domain containing protein 1*	6	5	1.00	4	12	1.00
5873	19923264	**RAB27A**	*RAB27A, member RAS oncogene family*	5	6	1.00	6	5	1.00
23516	190358541	**SLC39A14**	*solute carrier family 39 member 14*	6	5	1.00	8	7	1.00
10653	10863909	**SPINT2**	*serine peptidase inhibitor, Kunitz type, 2*	6	5	1.00	11	7	1.00
214	343168770	**ALCAM**	*activated leukocyte cell adhesion molecule*	8	3	0.99	3	5	0.99
84141	14149865	**FAM176A**	*eva-1 homolog A, regulator of programmed cell death*	5	5	1.00		3	
94005	15088795	**PIGS**	*phosphatidylinositol glycan anchor biosynthesis class S*	7	3	0.99	9	7	1.00
7355	109948265	**SLC35A2**	*solute carrier family 35 member A2*	7	3	0.99	5	4	1.00
54867	134152683	**TMEM214**	*transmembrane protein 214*	7	3	0.99	4	4	1.00
116068	84370276	**LYSMD3**	*LysM domain containing 3*	5	4	1.00	4		
334	214010181	**APLP2**	*amyloid beta precursor like protein 2*	4	4	1.00	4	7	1.00
6574	31543630	**SLC20A1**	*solute carrier family 20 member 1*	3	5	0.99	4		
79796	118026921	**ALG9**	*ALG9, alpha-1,2-mannosyltransferase*	6	2	0.97	8		
8879	31982936	**SGPL1**	*sphingosine-1-phosphate lyase 1*	2	6	0.97	4	6	1.00
252839	7705999	**TMEM9**	*transmembrane protein 9*	2	6	0.97	3		
9321	190194412	**TRIP11**	*thyroid hormone receptor interactor 11*	2	6	0.97		2	
199953	262399371	**TMEM201**	*transmembrane protein 201*	3	4	0.99			

23230	15619008	**VPS13A**	*vacuolar protein sorting 13 homolog A*			2	2	0.93	5		
347902	219521831	**AMIGO2**	*adhesion molecule with Ig-like domain 2*						4	4	1.00
382	4502211	**ARF6**	*ADP ribosylation factor 6*	7	7	38	19		33	23	1.00
51272	149192862	**BET1L**	*Bet1 golgi vesicular membrane trafficking protein like*				6		8	8	1.00
10384	261490684	**BTN3A3**	*butyrophilin subfamily 3 member A3*						4	7	1.00
56262	187829871	**LRRC8A**	*leucine rich repeat containing 8 family member A*			3			5	6	1.00
93380	27735037	**MMGT1**	*membrane magnesium transporter 1*						11	6	1.00
51594	41393547	**NBAS**	*neuroblastoma amplified sequence*						4	4	1.00
8613	29171740	**PPAP2B**	*phospholipid phosphatase 3*			9			9	7	1.00
5874	5729997	**RAB27B**	*RAB27B, member RAS oncogene family*			7			12	7	1.00
285282	62751417	**RABL3**	*RAB, member of RAS oncogene family like 3*	5	5	27	7		22	28	1.00
81539	117168275	**SLC38A1**	*solute carrier family 38 member 1*				4		5	5	1.00
55627	102467481	**SMPD4**	*sphingomyelin phosphodiesterase 4*	3	2	6	13		26	18	1.00
81848	188595697	**SPRY4**	*sprouty RTK signaling antagonist 4*	5	4	32	8		37	23	1.00
55240	56549145	**STEAP3**	*STEAP3 metalloreductase*	9	7	37	11		50	35	1.00
23670	209447096	**TMEM2**	*transmembrane protein 2*	3	2	11	9		16	13	1.00
9528	20070191	**TMEM59**	*transmembrane protein 59*			4			7	6	1.00
101	256773264	**ADAM8**	*ADAM metallopeptidase domain 8*						3	6	0.99
116254	20302038	**C6orf72**	*glycoprotein integral membrane 1*				3		4	3	0.99
54901	93277076	**CDKAL1**	*CDK5 regulatory subunit associated protein 1 like 1*	17	15	55	34		56	45	0.99
11261	6005731	**CHP**	*calcineurin like EF-hand protein 1*			4			3	5	0.99
1605	294997282	**DAG1**	*dystroglycan 1*			13			13	3	0.99
115817	19923983	**DHRS1**	*dehydrogenase/reductase 1*						3	5	0.99
2010	4557553	**EMD**	*emerin*	22	21	87	52		100	59	0.99
91289	255918129	**LMF2**	*lipase maturation factor 2*	7	5	17	10		21	20	0.99
54884	203098013	**RETSAT**	*retinol saturase*			3			3	4	0.99
9517	4758668	**SPTLC2**	*serine palmitoyltransferase long chain base subunit 2*			6			4	3	0.99
1186	14149607	**CLCN7**	*chloride voltage-gated channel 7*	10	9	40	15		42	28	0.98
9445	11527402	**ITM2B**	*integral membrane protein 2B*	3		25	9		25	12	0.98
129401	31982904	**NUP35**	*nucleoporin 35*			2			2	6	0.97
5327	14702169	**PLAT**	*plasminogen activator, tissue type*						2	4	0.97

10066	5730031	**SCAMP2**	*secretory carrier membrane protein 2*				6	2	6	0.97
79939	164607128	**SLC35E1**	*solute carrier family 35 member E1*	3	2	11	5	10	11	0.97
9166	37694065	**EBAG9**	*estrogen receptor binding site associated, antigen, 9*					2	3	0.96
51075	221136993	**TMX2**	*thioredoxin related transmembrane protein 2*			2		3	2	0.96
3710	153945846	**ITPR3**	*inositol 1,4,5-trisphosphate receptor type 3*				19	7	21	0.95
26608	7549793	**TBL2**	*transducin (beta)-like 2*					2	2	0.94
79143	225703079	**MBOAT7**	*membrane bound O-acyltransferase domain containing 7*	24	2	106		119	56	0.92
57130	170016077	**ATP13A1**	*ATPase 13A1*	11	6	28	21	32	25	0.91
1528	41281768	**CYB5A**	*cytochrome b5 type A*	4		9	6	14	13	0.91
