# Supplementary material for: The interactome of metabolic enzyme carbonic anhydrase IX reveals novel roles in tumor cell migration and invadopodia/MMP14-mediated invasion
Source: Oncogene. 2017 Jul 10;36(45):6244–61. doi: 10.1038/onc.2017.219 (PMC5684442; doi:10.1038/onc.2017.219)
Supplement: Supplementary Figures and Legends [file onc2017219x2.doc]

**Supplementary Figures and legends**

**
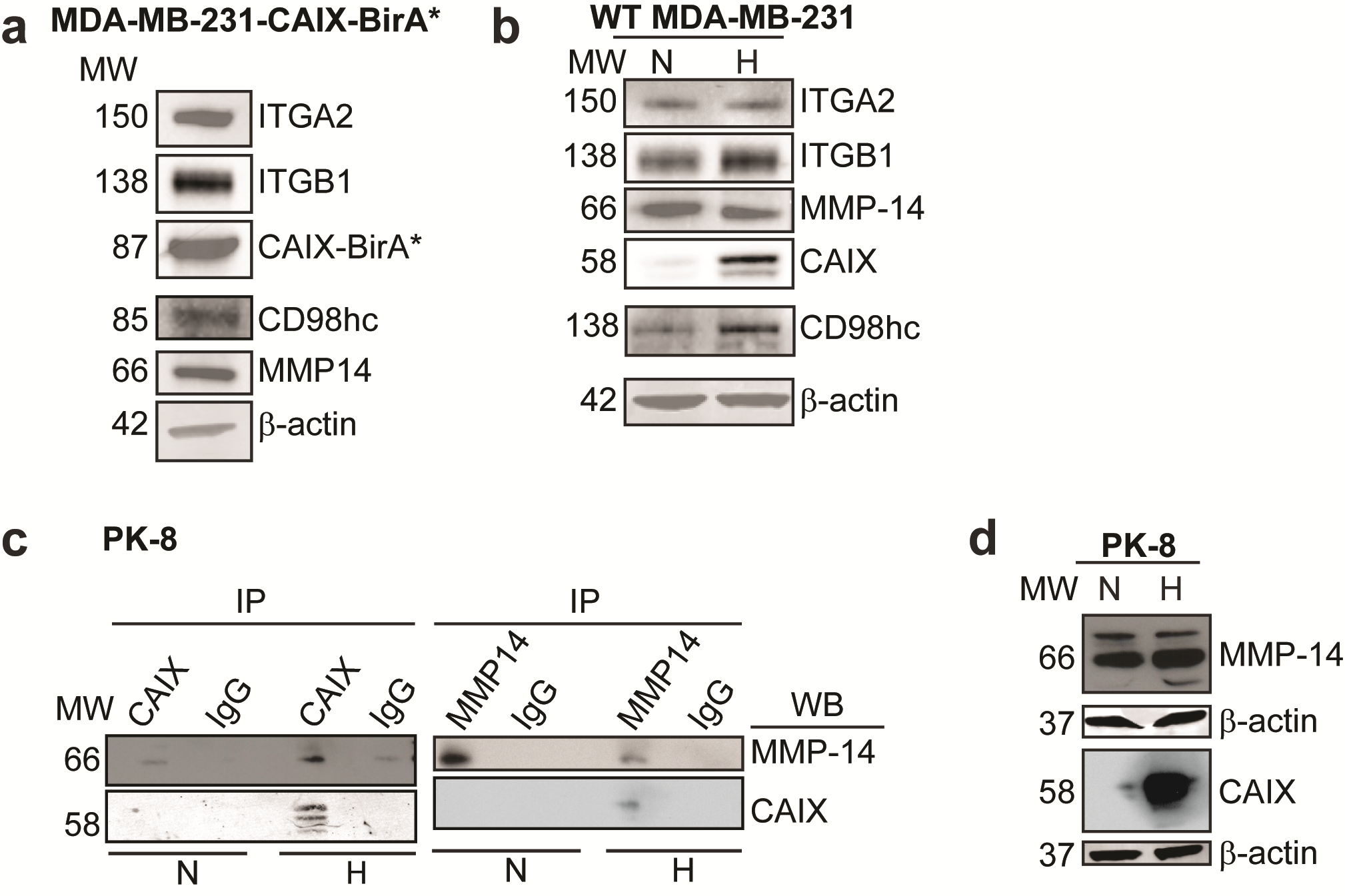
**

**Supplementary Figure S1. Levels of protein expression in the cell lysates used for co-immunoprecipitations in Figure 1.** (**a** and **b**) Western blots of cell lysates used for co-IPs showing the levels of expression of ITGA2, ITGB1, CD-98hc, MMP-14 and CAIX by (**a**) MDA-MB-231-CAIX-BirA* cells constitutively expressing the CAIX-BirA* fusion protein in normoxia and (**b**) WT MDA-MB-231 cells cultured in normoxia (N) or hypoxia (H). (**c**) Reciprocal co-IP of CAIX and MMP-14 from PK-8 human pancreatic ductal adenocarcinoma (PDAC) cells cultured in normoxia (N) or hypoxia (H). (**d**) Western blots of cell lysates used for co-IPs in panel C showing the levels of expression of CAIX and MMP-14 in PK-8 cells cultured in normoxia (N) or hypoxia (H).

**
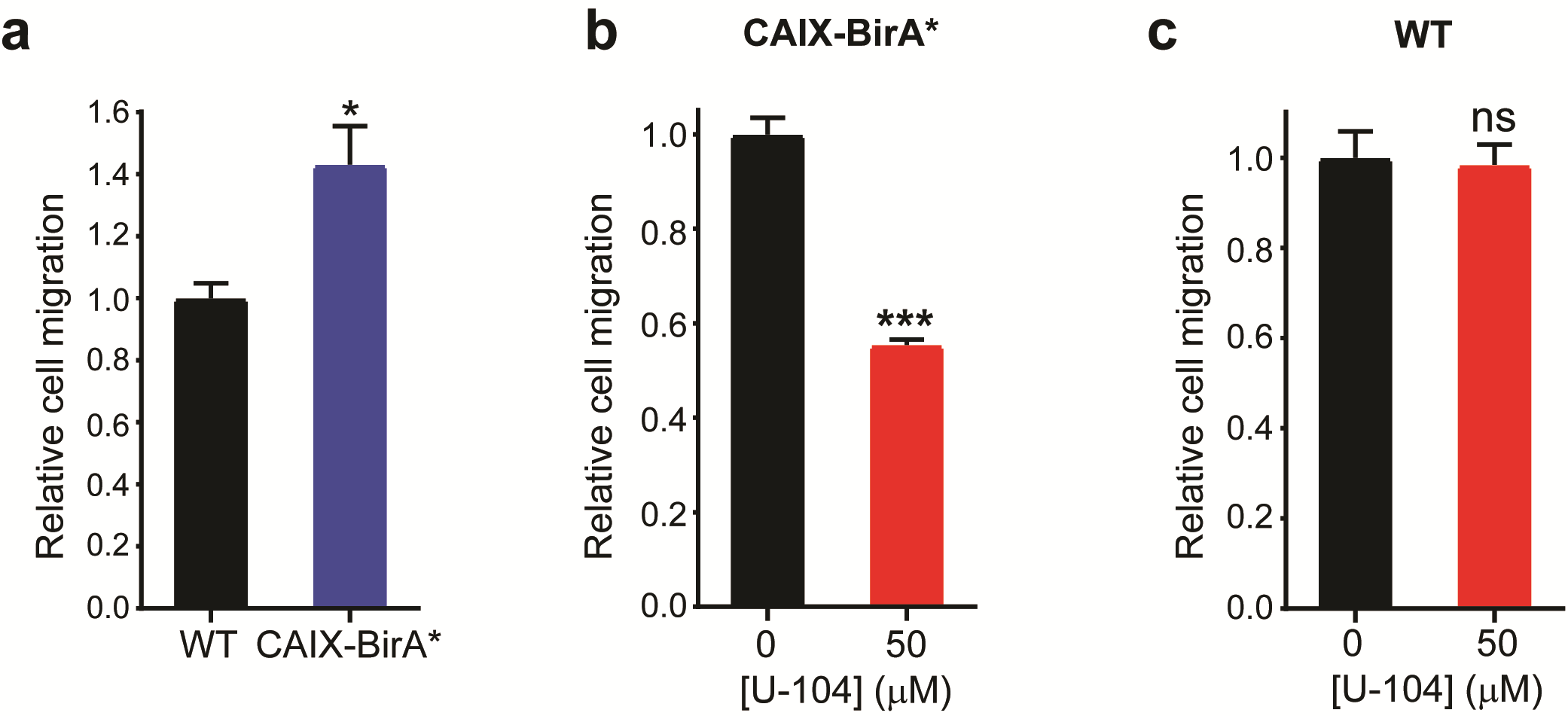
**

**Supplementary Figure S2**. **Chemotaxis migration assay demonstrating the role of CAIX in migration of breast cancer cells.** (**a**) Migration through transwell filters by the indicated MDA-MB-231 cell lines cultured in normoxia for 24 hours. Data show the mean ± SEM of technical replicates (n=6) and are representative of 2 independent experiments. **P*<0.05. (**b** and **c**) The relative change in migration by (**b**) MDA-MB-231 CAIX-BirA* cells and (**c**) WT MDA-MB-231 cells cultured in normoxia for 24 hours in the presence or absence of 50µM U-104. Data show the mean ± SEM of technical replicates (n=8) and are representative of 2 independent experiments. **P*<0.05, ****P*<0.001.

Statistical analysis was performed using Student’s *t*-test.

**
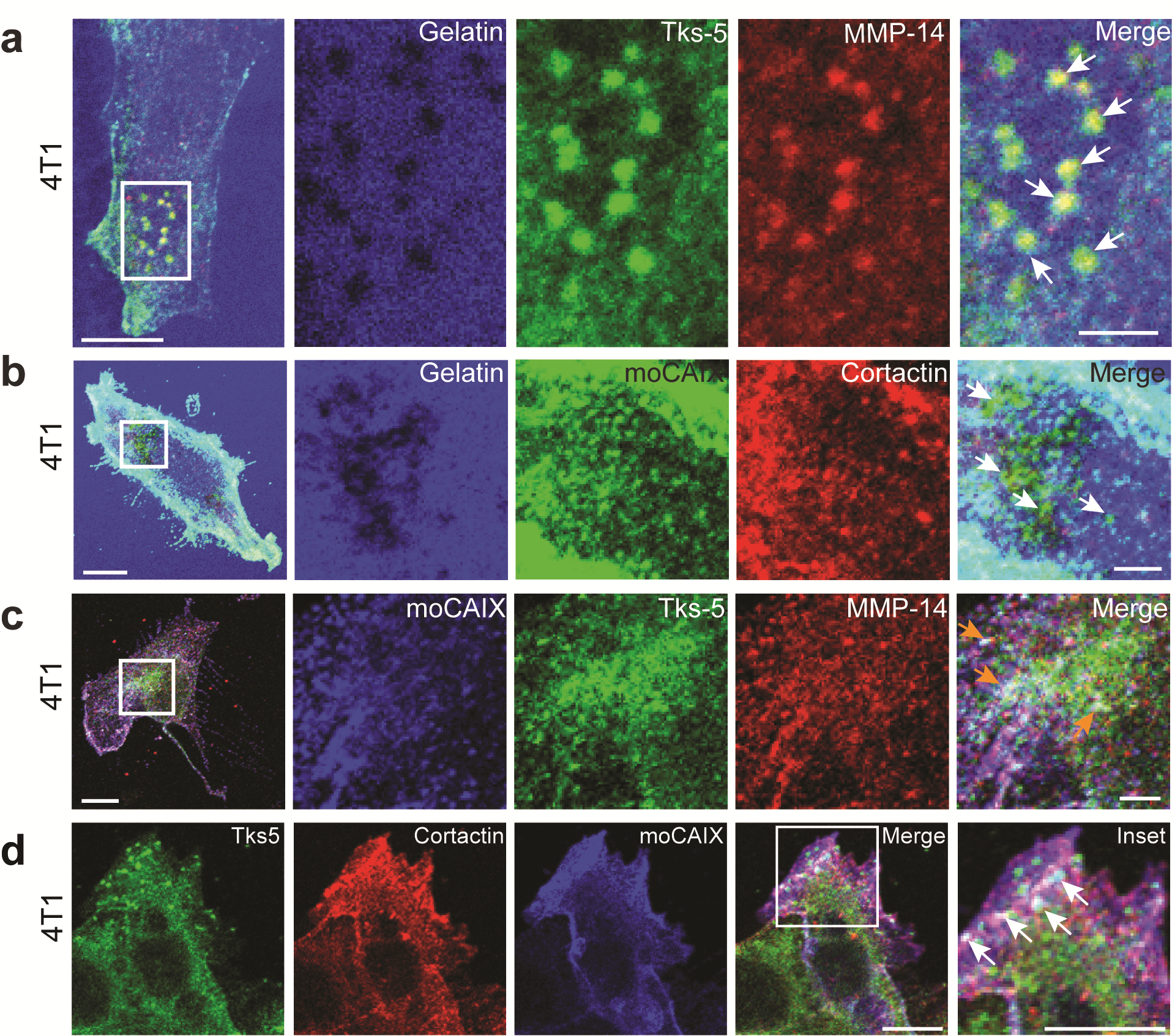
**

**Supplementary Figure S3. Co-localization of CAIX with MMP-14 at the invadopodia of 4T1 cells in hypoxia.** (**a**) Images showing parental 4T1 cells cultured in hypoxia on fluorescently-labeled gelatin (blue, with black areas denoting regions of invadopodia-mediated degradation) and stained for Tks-5 (green) and MMP-14 (red) to demonstrate co-localization at active invadopodia (arrows; yellow foci). Scale bar, 10 μm. (**b**) Images showing parental 4T1 cells cultured as described in A and stained for moCAIX (green) and cortactin (red) to demonstrate localization of CAIX at active invadopodia (arrows). Scale bar, 10 μm. (**c**) Images showing co-localization of CAIX (blue), Tks-5 (green) and MMP-14 (red) at invadopodia (orange arrows; white foci) formed by parental 4T1 cells cultured in hypoxia. Scale bar, 10 μm. (**d**) Images showing co-localization of moCAIX (blue), Tks-5 (green) and Cortactin (red) at invadopodia (white arrows; white foci) formed by parental 4T1 cells cultured in hypoxia. Scale bar, 10 μm.

**
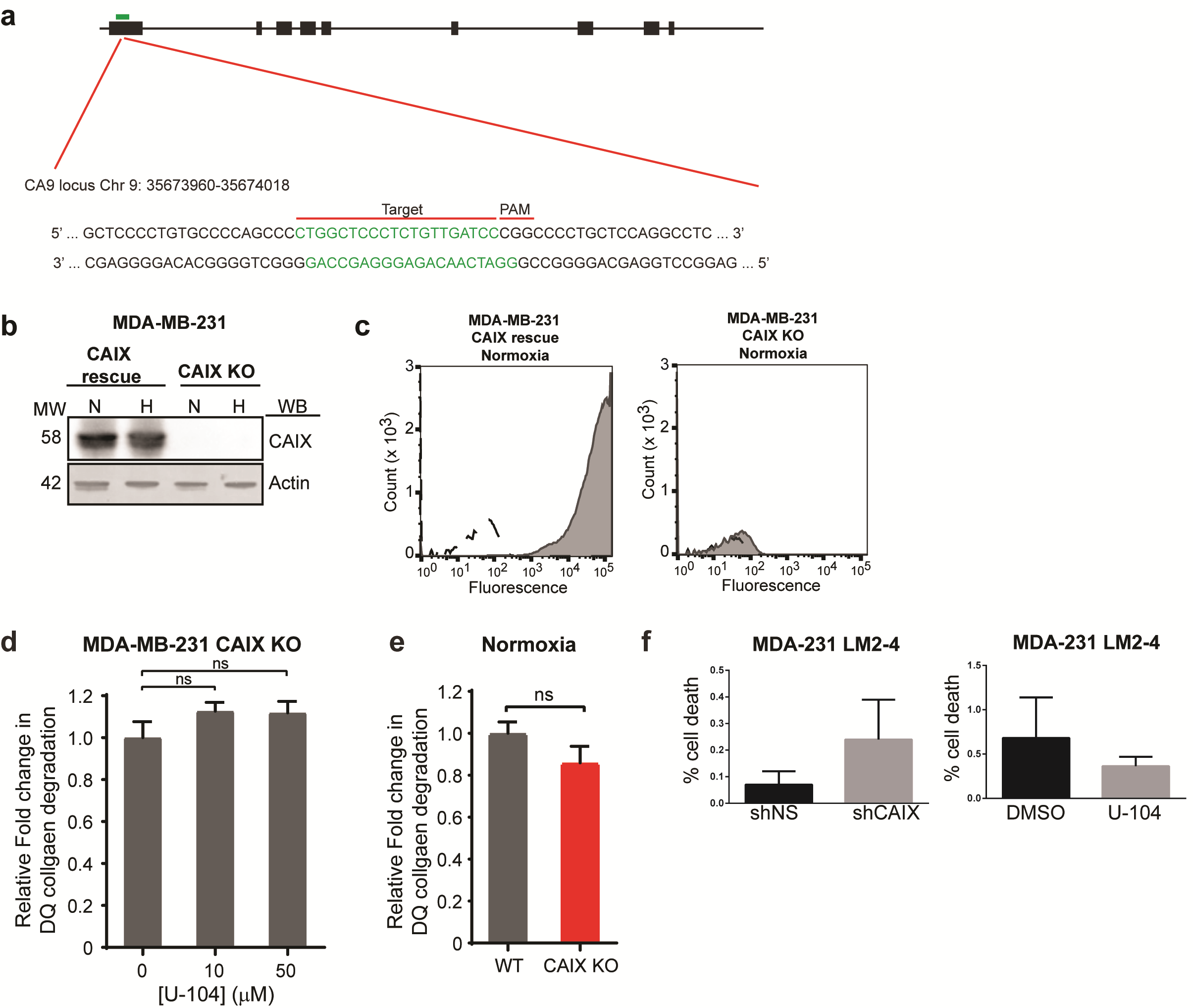
**

**Supplementary Figure S4. Characterization of CAIX KO and CAIX rescue cell lines.** (**a**) Schematic outlining Cas9-mediated disruption of the *CA9* locus. The sgRNA target sequence is shown in green. (**b**) Western Blot showing the expression of CAIX by the indicated MDA-MB-231 cell lines grown in either normoxia (N) or hypoxia (H). β-actin was used as a loading control. (**c**) FACS analysis showing CAIX expression by the MDA-MB-231 CAIX KO and CAIX rescue cell lines cultured in normoxia. The filled (gray) histograms show specific CAIX staining and the dotted lined histograms show isotype specific IgG staining. (**d**) Analysis of DQ collagen degradation by MDA-MB-231 CAIX KO cells treated with U-104. Data show the mean ± SEM of technical replicates (n=4) and are representative of 2 independent experiments. (**e**) Analysis of DQ collagen degradation by the indicated MDA-MB-231 cell lines cultured in normoxia. Data show the mean ± SEM of technical replicates (n=5) and are representative of 2 independent experiments. (**f**) Analysis of cell death as detected by TUNEL for the indicated cell lines as a control for the invasion assays described in Figure 4 H and I. Results from a representative experiment are presented showing the mean ± SEM of 3 random fields per sample.


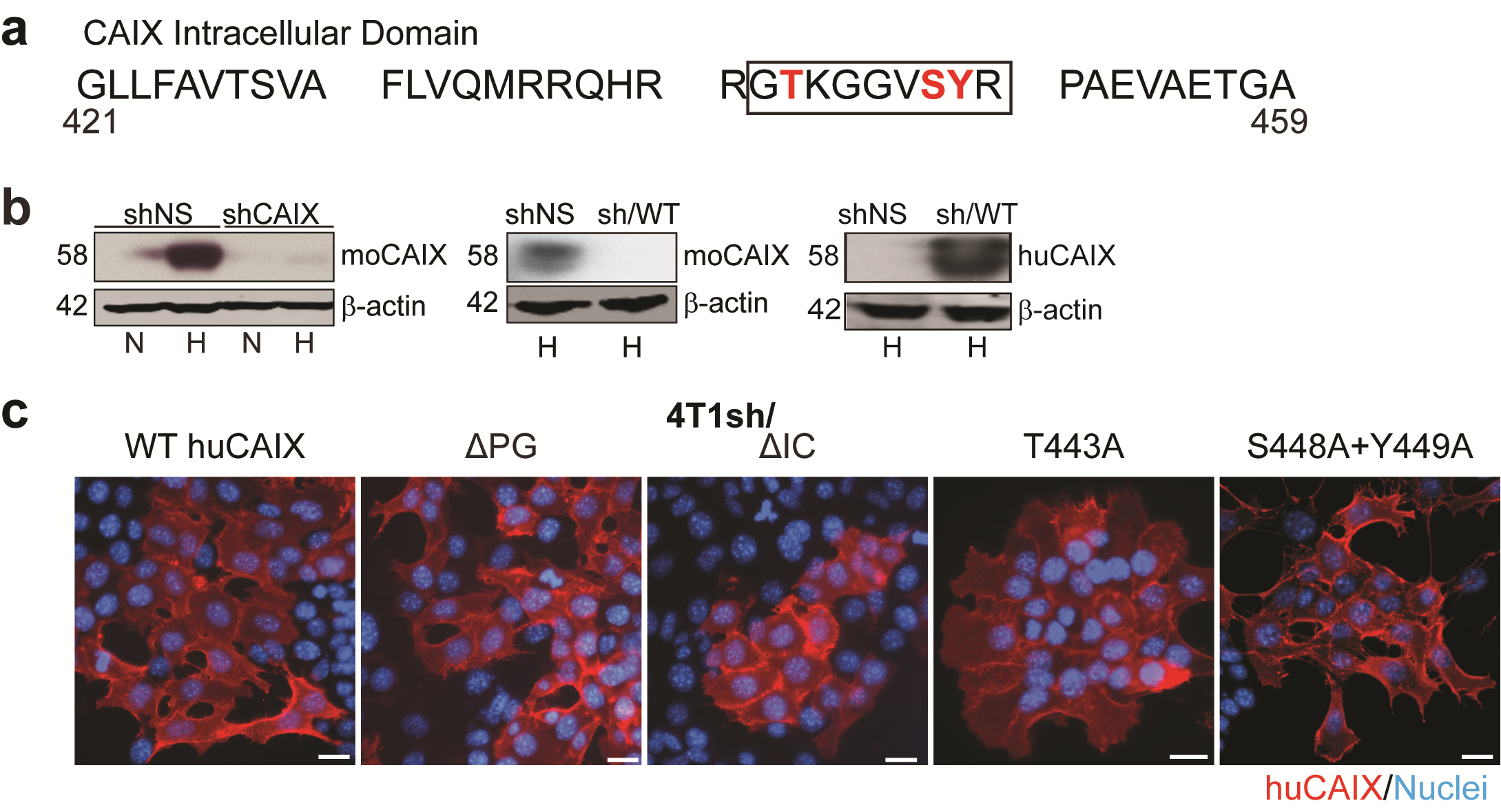


**Supplementary Figure S5. Characterization of the 4T1shCAIX cell lines overexpressing huCAIX variants.** (**a**) Protein sequence for the IC domain of CAIX showing the region containing putative phosphorylation sites (black box) and the residues targeted for site-directed mutagenesis (red). (**b**) Western blots showing CAIX expression by 4T1 cells expressing non-silencing shRNA (shNS), shRNA targeting mouse CAIX (shCAIX) or shCAIX paired with mouse shCAIX-resistant wild-type (WT) human CAIX (sh/WT) cultured in either normoxia (N) or hypoxia (H). (**c**) Images showing cell surface expression of the indicated huCAIX constructs (red) by cells cultured in hypoxia. Scale bar, 20 μm.

**
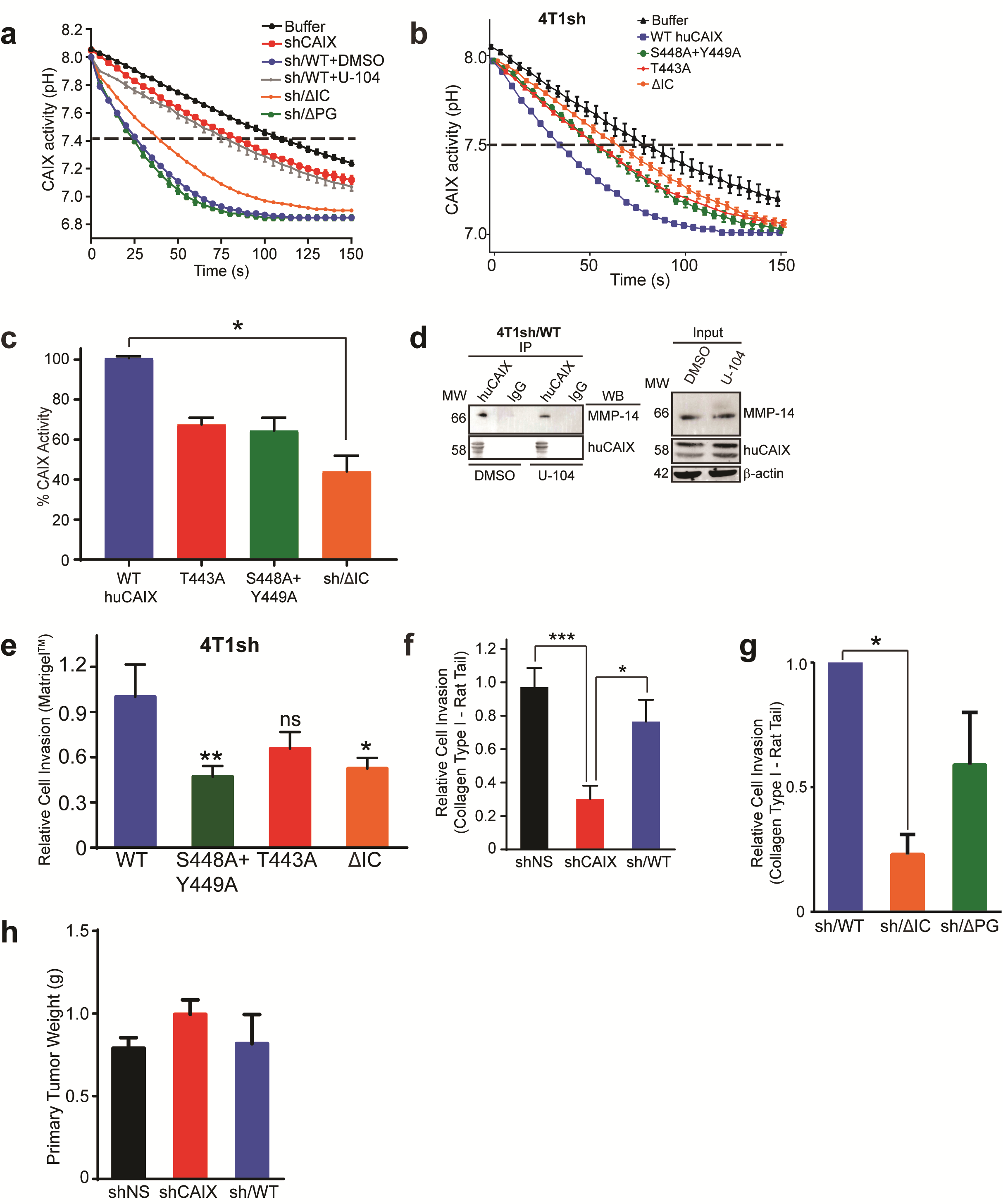
**

**Supplementary Figure S6. Analysis of cell-based CAIX activity and invasion by 4T1 cells expressing variants of CAIX.** (**a-b**) Analysis of CAIX catalytic activity using the *in-cell* Carbonic Anhydrase activity assay. The decrease in the pH of Tris-HCl buffer containing the indicated 4T1 cell lines was monitored following addition of saturated CO_2_ solution. Assays were performed in normoxia in the presence or absence of U-104 (50 μM) as indicated. Data show the mean ± SEM of technical replicates (n=3/group) and are representative of 3 independent experiments. (**c**) Levels of CAIX catalytic activity in panel b were normalized by using the time required to achieve 50% of the total decrease in pH. Data were normalized to the spontaneous rate of reaction in the presence of buffer alone and the activity of cells expressing WT huCAIX was set to 100%. Data show the mean ± SEM of technical replicates (n=3/group) and are representative of 3 independent experiments **P*<0.05, ****P*<0.001. (**d**) Co-IP analysis of the interaction between huCAIX and MMP-14 in 4T1 cells cultured in hypoxia and treated with U-104 (50 μM). Isotype-specific IgG was used as a control Ab for the Co-IPs. Western blots showing the levels of expression of huCAIX and MMP-14 in the lysates of 4T1 cells cultured in hypoxia and used for the Co-IPs are shown to the right. β-actin was used as a loading control. (**e**) Analysis of cell invasion through Matrigel^TM^ in hypoxia by the indicated 4T1 cell lines. **P*<0.05, ***P*<0.01. Statistical analysis was performed using ANOVA. (**f**) Invasion through type 1 collagen by the indicated 4T1 cell lines cultured in hypoxia. **P*<0.05, ****P*<0.001. (**g**) Invasion through type 1 collagen by the indicated 4T1 cell lines cultured in hypoxia. **P*<0.05, ****P*<0.001. Data in (**e**) to (**g**) show the mean ± SEM of 3 independent experiments. (**h**) Primary tumor size, expressed as weight in grams, from mice harvested for assessment of lung metastases. Data are presented as mean ± SEM. n=6/group.


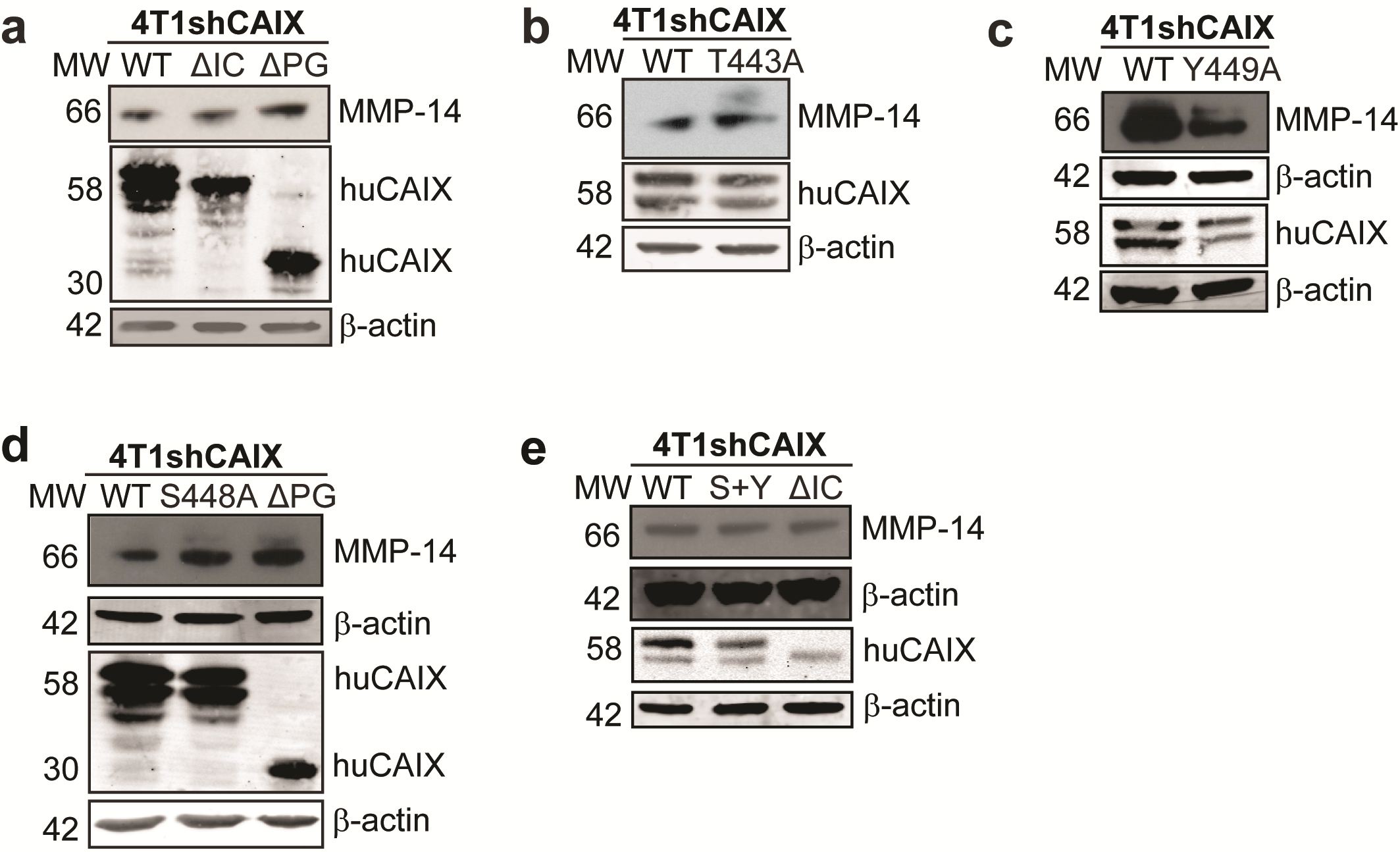


**Supplementary Figure S7. Levels of protein expression in the cell lysates used for co-immunoprecipitations in Figure 6.** (**a** to **e**) Western blots showing the levels of expression of huCAIX and MMP-14 in 4T1shCAIX cells overexpressing (**a**) WT huCAIX, ΔIC, ΔPG truncation variants, and huCAIX containing the point mutations (**b**) T443A, (**c**) Y449A, (**d**) S448A, and (**e**) S448A + Y449A that were used for co-IPs. β-actin was used as a loading control.

**
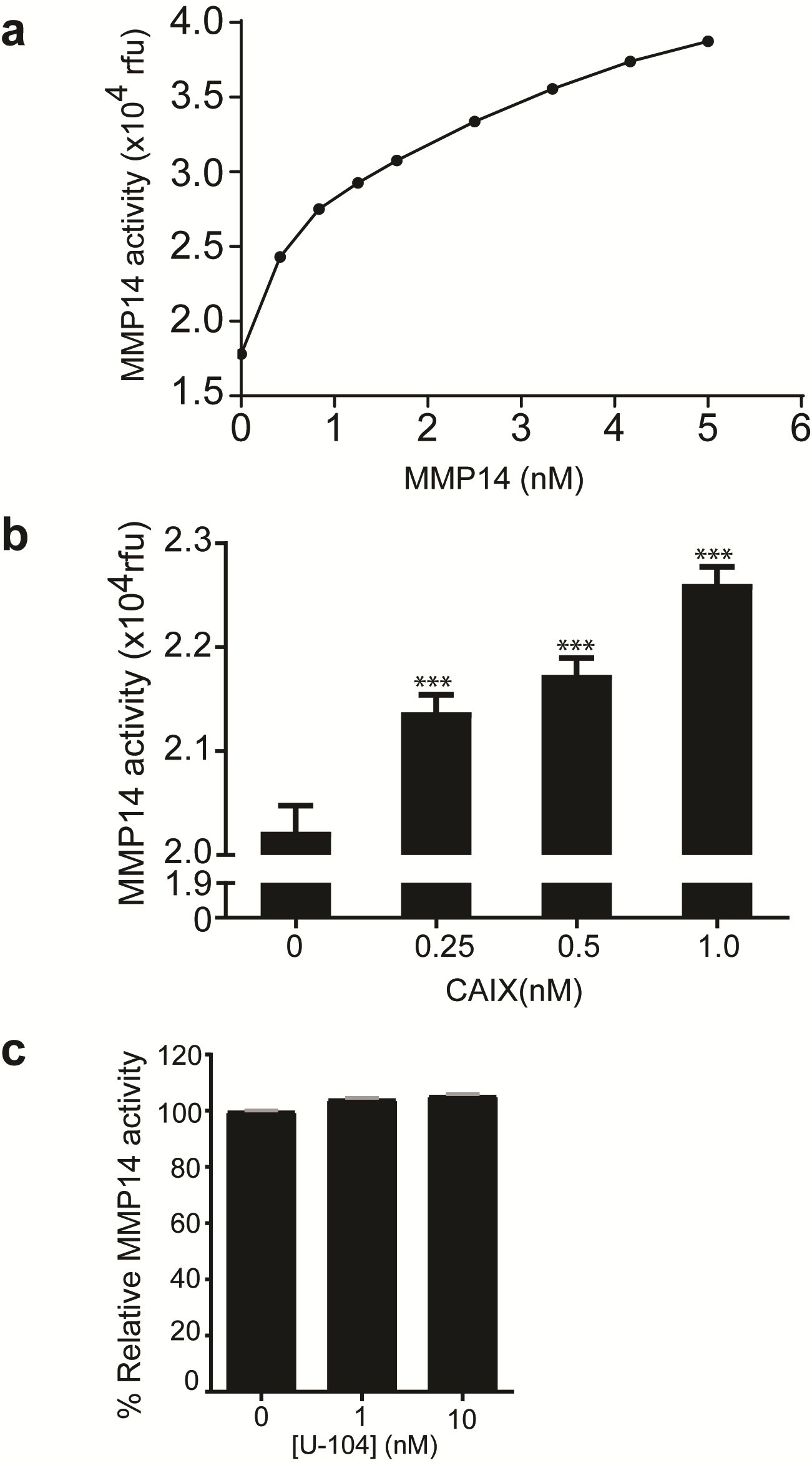
**

**Supplementary Figure S8. Evaluation of CAIX-mediated stimulation of MMP-14 activity**. (**a**) Graph showing the MMP-14 activity in response to increasing concentrations of MMP-14. Data show the mean ± SEM of technical replicates, n = 5. **P*<0.05, ****P*<0.001. (**b**) MMP-14 activity in the presence of increasing concentrations of CAIX. Data show the mean ± SEM of technical replicates (n=5) and are representative of 3 independent experiments. ****P*<0.001. (**c**) Relative MMP-14 activity in the presence of increasing concentrations of the specific small molecule inhibitor of CAIX, U-104. Data show the mean ± SEM of technical replicates (n=5).

**Supplementary movie legends**

**Supplementary Movie S1:** A time lapse video consisting of images taken every 2 hours over a period of 24 hours showing wound-induced cell migration by MDA-MB-231-CAIX-BirA* cells cultured in normoxia in the absence of U-104 (DMSO control). The images were taken using an Incucyte ZOOM live cell analysis instrument with a Nikon Plan Fluor 10x/0.3 NA objective in bright field mode. Scale bar, 300µm.

**Supplementary Movie S2:** A time lapse video consisting of images taken every 2 hours over a period of 24 hours showing wound-induced cell migration by MDA-MB-231-CAIX-BirA* cells cultured in normoxia in the presence of 75µM U-104. The images were taken using an Incucyte ZOOM live cell analysis instrument with a Nikon Plan Fluor 10x/0.3 NA objective in bright field mode. Scale bar, 300µm.
